# Macrophage Replication Screen Identifies a Novel *Francisella* Hydroperoxide Resistance Protein Involved in Virulence

**DOI:** 10.1371/journal.pone.0024201

**Published:** 2011-09-06

**Authors:** Anna C. Llewellyn, Crystal L. Jones, Brooke A. Napier, James E. Bina, David S. Weiss

**Affiliations:** 1 Department of Microbiology and Immunology, Microbiology and Molecular Genetics Program, Emory University, Atlanta, Georgia, United States of America; 2 Emory Vaccine Center, Emory University, Atlanta, Georgia, United States of America; 3 Department of Microbiology, Immunology and Biochemistry, University of Tennessee Health Science Center, Memphis, Tennessee, United States of America; 4 Division of Infectious Diseases, Department of Medicine, Emory University, Atlanta, Georgia, United States of America; Universidad Nacional, Costa Rica

## Abstract

*Francisella tularensis* is a Gram-negative facultative intracellular pathogen and the causative agent of tularemia. Recently, genome-wide screens have identified *Francisella* genes required for virulence in mice. However, the mechanisms by which most of the corresponding proteins contribute to pathogenesis are still largely unknown. To further elucidate the roles of these virulence determinants in *Francisella* pathogenesis, we tested whether each gene was required for replication of the model pathogen *F. novicida* within macrophages, an important virulence trait. Fifty-three of the 224 genes tested were involved in intracellular replication, including many of those within the *Francisella* pathogenicity island (FPI), validating our results. Interestingly, over one third of the genes identified are annotated as hypothetical, indicating that *F. novicida* likely utilizes novel virulence factors for intracellular replication. To further characterize these virulence determinants, we selected two hypothetical genes to study in more detail. As predicted by our screen, deletion mutants of *FTN_0096* and *FTN_1133* were attenuated for replication in macrophages. The mutants displayed differing levels of attenuation *in vivo*, with the *FTN_1133* mutant being the most attenuated. *FTN_1133* has sequence similarity to the organic hydroperoxide resistance protein Ohr, an enzyme involved in the bacterial response to oxidative stress. We show that FTN_1133 is required for *F. novicida* resistance to, and degradation of, organic hydroperoxides as well as resistance to the action of the NADPH oxidase both in macrophages and mice. Furthermore, we demonstrate that *F. holarctica* LVS, a strain derived from a highly virulent human pathogenic species of *Francisella*, also requires this protein for organic hydroperoxide resistance as well as replication in macrophages and mice. This study expands our knowledge of *Francisella*'s largely uncharacterized intracellular lifecycle and demonstrates that FTN_1133 is an important novel mediator of oxidative stress resistance.

## Introduction

*Francisella tularensis* is a unique facultative intracellular pathogen that can cause a potentially lethal disease with an infectious dose as low as 10 bacteria [1]. A small Gram-negative coccobacillus, *F. tularensis* is the causative agent of tularemia, a vector- and water-borne zoonotic disease resulting in non-specific, flu-like symptoms that may culminate in pneumonic, glandular, and systemic infections [1]. When left untreated, pneumonic tularemia can result in a mortality rate as high as 60% [2]. *F. tularensis* subspecies are endemic across the Northern Hemisphere, with the majority of reported cases of disease in the United States, Europe, Russia, and Japan [3]. Due to its extreme infectivity, high morbidity and mortality rates, history of weaponization, and ease of aerosolization, dissemination, and genetic manipulation, *F. tularensis* is considered a category A potential bioweapon by the Centers for Disease Control and Prevention (CDC) [4]–[6].

The virulence mechanisms of *F. tularensis* subspecies and other *Francisella* species are still being characterized, including the role of the *Francisella* pathogenicity island (FPI) which is thought to encode a Type VI secretion system that facilitates the release of virulence proteins into host cells [7]–[13]. While *F. tularensis* and *F. holarctica* are responsible for the majority of disease burden in humans [5], many important virulence determinants are conserved among other *Francisella* species, including *F. novicida*. In addition to the FPI, these conserved virulence determinants include the presence of a non-inflammatory lipopolysaccharide (LPS), protective capsule, siderophores, and proteins involved in resistance to oxidative stress [14]–[21]. *F. novicida*, which has 98% nucleotide identity with the human pathogenic species, causes disease mainly in immunocompromised individuals but has also been shown to cause disease in healthy individuals [22]–[24]. The live vaccine strain (LVS) is an attenuated strain of the highly pathogenic species *F. holarctica* that was originally developed as a vaccine and retains 99.92% identity to its parental species [25], [26]. Though work involving *F. tularensis* and *F. holarctica* is restricted to Select Agent Biosafety Level 3 (BSL3) laboratories, both *F. novicida* and LVS are approved for use in BSL2 laboratories, are readily genetically manipulated, and cause tularemia-like disease in mice, making them both good laboratory models for studying *Francisella* pathogenesis [27], [28].

*Francisella*'s primary replicative niche is thought to be the cytosolic compartment of both phagocytic and non-phagocytic cells such as macrophages, neutrophils, hepatocytes, alveolar epithelial cells, and fibroblasts [29]–[32]. After being engulfed by phagocytic host cells, the bacteria are taken up into phagosomes where they are confronted with a myriad of antimicrobial defenses including degradative enzymes, acidic pH, and oxidative stress [17]–[21], [33]–[36]. The reactive oxygen species (ROS) which cause oxidative stress can directly damage bacterial macromolecules such as proteins, DNA, and lipids. They can also react with these macromolecules to generate more ROS and toxic oxygen compounds, including the highly toxic organic hydroperoxides that result from the destructive lipid peroxidation of cell membranes [37]–[40]. Within host cells, ROS are generated by multiple mechanisms including the NADPH oxidase, myeloperoxidase, lipoxygenases, and cellular respiration [41], [42]. The NADPH oxidase, which produces superoxide radicals that lead to ROS formation, has been shown to be important for the host response to infection with *Francisella* species [43]–[46]. These bacteria employ numerous strategies to resist oxidative stress including limiting the activation of the NADPH oxidase [35], [36], [47] and using multiple enzyme systems to detoxify reactive oxygen compounds [18], [32], [48], [49].

While a general outline of *Francisella*'s interaction with host cells is known, the specific mechanisms of cell entry, phagosomal escape, cytosolic replication, and some of the ways it evades immune defenses are still unknown. Genome-wide *in vivo* screens have identified genes required for the virulence of several *Francisella* species but do not shed light on how the majority of these genes contribute to pathogenesis [50]–[52]. As replication within host cells is a major part of *Francisella*'s infectious cycle, we set out to determine which of the genes that are known to be required for virulence *in vivo* are also required for replication in host macrophages.

We performed an intracellular replication screen using transposon mutants representing 224 genes that have previously been shown to be required for virulence *in vivo*. Fifty-three of the genes tested were required for replication in macrophages including many of the FPI genes, validating the screen. We also identified biotin biosynthetic genes and the *fsl*/*fig* siderophore biosynthetic genes [16], [53]–[55] as well as numerous proteins of unknown function as being required for replication in macrophages. We validated the intracellular and *in vivo* requirement of two of these novel genes, *FTN_1133* and *FTN_0096*. We then further investigated the importance of *FTN_1133*, which encodes a protein with sequence similarity to Ohr, a protein involved in oxidative stress resistance [56]–[64]. Accordingly, we find that FTN_1133 is required for resistance to, and degradation of, organic hydroperoxides. Furthermore, the replication defect of the *FTN_1133* mutant is rescued in macrophages lacking a functional NADPH oxidase and partially rescued in mice with the same defect. We further demonstrate that *F. holarctica* LVS also requires this protein for organic hydroperoxide resistance and replication in macrophages and mice. Taken together, these data highlight the critical role that novel virulence factors play in *Francisella* pathogenesis and contribute to the elucidation of the requirements for this pathogen's largely uncharacterized intracellular lifecycle.

## Materials and Methods

### Ethics Statement

All experimental procedures were approved by the Emory University Institutional Animal Care and Use Committee (protocol #069-2008Y).

### Bacterial strains and growth conditions

Wild-type *F. novicida* strain U112, a previously described *mglA* point mutant, GB2 [65], and the *F. holarctica* Live Vaccine Strain (LVS) were generous gifts from Dr. Denise Monack (Stanford University, Stanford, CA). *F. novicida* overnight cultures were grown at 37°C on a rolling drum in tryptic soy broth (TSB; Difco/BD, Sparks, MD) supplemented with 0.02% L-cysteine (Sigma-Aldrich, St. Louis, MO) while LVS cultures were grown in modified Mueller-Hinton broth (mMHB) supplemented with 1 mM CaCl_2_, 1 mM MgCl_2_, 0.1% glucose (Sigma-Aldrich), 2% Isovitalex (Difco/BD), and 0.025% ferric pyrophosphate as previously described [66]. Growth in minimal medium was determined using Chamberlain's chemically defined minimal medium, prepared as previously described [67]. For the replication screen, *F. novicida* was plated for enumeration on tryptic soy agar (TSA; Difco/BD) and supplemented with 0.01% L-cysteine. Bacteria from all other *F*. *novicida* experiments were plated on modified Mueller Hinton (mMH) (Difco/BD) plates supplemented with 0.025% ferric pyrophosphate (Sigma-Aldrich), 0.1% glucose, and 0.01% L-cysteine. LVS was plated on mMH supplemented additionally with 2% Isovitalex. When appropriate, kanamycin (Kan; Fisher Scientific, Fair Lawn, NJ) was added to media at a concentration of 30 µg/ml for *F. novicida* and 10 µg/ml for LVS.

### Macrophages

RAW264.7 murine macrophages (ATCC, Manassas, VA) were cultured in Dulbecco's modified Eagle medium (high glucose, L-glutamine; DMEM; Lonza, Walkersville, MD) supplemented with 10% heat-inactivated fetal calf serum (FCS; HyClone, Logan, UT). Bone marrow-derived macrophages (BMM) were isolated from either wild-type C57BL/6 or gp91^phox-/-^ mice (Jackson Laboratories, Bar Harbor, ME) and cultured as described previously [68] in DMEM supplemented with 10% heat-inactivated FCS and 10% macrophage colony-stimulating factor (M-CSF)-conditioned medium (collected from M-CSF-producing L929 cells). Macrophages were incubated before and during infection at 37°C with 5% CO_2_.

### Intracellular replication screen and macrophage infections

The screen library was assembled by inoculation of individual transposon mutants from the *F. novicida* two-allele transposon library [69] into 96-well plates containing cysteine-supplemented TSB. These were grown overnight at 37°C, glycerol (Fisher Scientific) was added to 20% final volume, and the plates were stored at −80°C. RAW264.7 murine macrophages were seeded at 10^5^ cells/well in 96-well tissue culture plates for the replication screen or 5×10^5^cells/well in 24-well tissue culture plates for subsequent infections and incubated overnight. The medium was then removed and the macrophages were infected with overnight cultures of individual mutants from the screen library that had been diluted in DMEM/10% FCS to achieve a multiplicity of infection (MOI) of twenty bacteria per macrophage. The plates were centrifuged for 15 minutes at 931 x g at room temperature and then incubated for 30 minutes. Next, the macrophages were washed twice with DMEM and incubated for an additional 30 minutes in DMEM/10% FCS containing 100 µg/ml of gentamicin (TekNova, Hollister, CA). The macrophages were again washed twice and DMEM/10% FCS with 10 µg/ml gentamicin was added. At 1 and 24 hours post-infection, the macrophages were washed twice and then lysed with 1% saponin (Alfa Aesar, Heysham, Lancs., UK) in phosphate buffered solution (PBS) without calcium and magnesium (Lonza, Walkersville, MD). Serial dilutions of the resulting macrophage lysates were plated onto cysteine-supplemented TSA in sterile 24-well plates. Finally, the colony forming units (CFU) for each transposon mutant were counted and the fold replication (CFU at 24 hr/ CFU at 1 hr) was calculated and compared to the fold replication of wild-type *F. novicida* U112. A similar infection procedure was followed for both wild-type and gp91^phox-/-^ BMM infections with the following modifications: 3×10^5^ BMM were plated per well in a 24-well plate, DMEM/10% FCS/10% M-CSF was used throughout and the final time point was 5.5 hours for *F. novicida* infections or 24 hours for LVS infections. The replication screen was performed twice and the data were averaged to determine the final results. All transposon mutants that replicated less than or equal to 30% of the wild-type value (mutant fold replication/wild-type fold replication ≤0.3) were considered attenuated for replication *in vitro.* Results for all transposon mutants tested are listed in Table S1.

### Mutagenesis and complementation

To generate *F. novicida* deletion mutants, PCR was used to amplify flanking DNA regions upstream and downstream of the gene of interest. A Kan-resistance cassette was sewn in between these flanking regions using overlapping PCR reactions. The final linear PCR products were then gel purified and transformed into chemically competent wild-type U112 as previously described [70]. The primers used to create the Kan-resistant deletion mutants contained FRT sites flanking the Kan-resistance cassette, which allowed removal of the cassette using the plasmid pFFlp encoding the Flp-recombinase as previously described [71]. Constructs for the complementation of each mutant were generated by overlapping PCR using PCR-amplified fragments of the wild-type gene of interest, upstream and downstream flanking regions, and a Kan-resistance cassette. These constructs were then transformed into the appropriate chemically competent deletion mutants. Verification of allelic replacement in mutant and complemented strains was performed using check primers in PCR reactions on purified genomic DNA from each strain. PCR products of the correct size were subsequently sequenced (MWG Operon, Huntsville, AL) for final verification of allelic replacement. For LVS mutagenesis, we employed both targeted gene disruption via group II introns as previously described [72] and allelic replacement. Briefly, in order to perform targeted gene disruption, primers for targeting the *FTL_0803* allele were generated using the TargeTron system (Sigma-Aldrich) and the resulting PCR product was cloned into the *Francisella* targeting vector, pKEK1140 (generously provided by Dr. Karl Klose, UTSA, San Antonio, TX) [72]. LVS was then transformed with the resulting vector and *FTL_0803* insertion mutants were selected for as previously described [72]. To generate LVS deletion mutants via allelic replacement, PCR was used to amplify flanking DNA regions upstream and downstream of the gene of interest which were then sewn together using overlapping PCR reactions. The final linear PCR products were then gel purified, digested with BamHI restriction enzyme (New England Biolabs, Ipswich, MA), dephosphorylated with Antarctic phosphatase (New England Biolabs), and ligated using T4 ligase (New England Biolabs) into the *Francisella* suicide vector pXB186 that encodes the *sacB* enzyme (James Bina, University of Tennessee, Memphis, TN). LVS was then transformed and mutants selected as previously described [73], [74]. Briefly, pXB186 ligations were transformed into LVS via electroporation and plated on chocolate agar with kanamycin (10 ug/ml). Next, kanamycin resistant colonies were plated on 10% sucrose, 1% hemoglobin cysteine heart agar (CHA) and surviving colonies were then patched onto kanamycin plates. Finally, genomic DNA from the kanamycin sensitive colonies was PCR-verified and sequenced to confirm the deletion. The *FTL_0803* targeted disruption mutant was used in the RAW264.7 LVS macrophage experiment and the *FTL_0803* clean deletion mutant was used in all other LVS experiments. Neither *FTN_1133* nor *FTL_0803* appear to be in an operon as the genes adjacent to both are transcribed in opposing directions. All primers used in this study are listed in Table S2.

### Mouse experiments

Female C57BL/6 and gp91^phox-/-^ mice (Jackson Laboratory, Bar Harbor, ME) between 7 and 10 weeks of age were kept under specific pathogen-free conditions in filter-top cages at Emory University and provided with sterile food and water *ad libitum*. Experimental studies were performed in accordance with the Emory University Institutional Animal Care and Use Committee guidelines. For competition experiments, mice were inoculated subcutaneously with a 1∶1 ratio of kanamycin-resistant deletion mutant and kanamycin-sensitive wild-type *F. novicida* for a total of 2×10^5^ CFU in 50 µl sterile PBS. For single infections, mice were infected with 2×10^5^ CFU subcutaneously. After 48 (*F. novicida* infections) or 72 hours (LVS infections), mice were sacrificed and the spleen, liver, and skin at the site of infection were harvested, homogenized (Tissue Tearor, Cole-Parmer, Vernon Hills, Illinois), plated for CFU on MH plates (with and without kanamycin for competition experiments), and then incubated overnight at 37°C. For single infections, organs were weighed before homogenization and the resulting CFU were divided by the weight of each organ to determine CFU/gram. For survival experiments, mice were infected as described for single strain infections and then observed for illness and sacrificed if they appeared moribund. For collection of RNA, mice were infected intraperitoneally with an infectious dose of 2×10^6^ CFU, four hours after which the mice were sacrificed and the livers collected for RNA isolation. Competitive index (CI) values were determined using the formula: (CFU mutant output/CFU WT output)/(CFU mutant input/CFU WT input).

### RNA isolation and quantitative real-time PCR

At various time points post-infection, BMM were lysed and homogenized in trizol reagent (MRC, Cincinnati, Ohio). Similarly, liver samples from intraperitoneally-infected mice (4 hours post-infection) were homogenized in trizol reagent. For both *in vitro* and *in vivo* samples, RNA was isolated using the RNeasy Mini kit (QIAGEN, Germantown, MD). Gene-specific primers (Table S2) were used to amplify *FTN_1133* transcripts using the Power Sybr Green One Step Kit (Applied Biosystems, Foster City, CA) on an Applied Biosystems StepOnePlus Real Time PCR System per the manufacturers' instructions. Expression of *FTN_1133* transcript was calculated relative to the expression of the DNA helicase *uvrD* (*FTN_1594*).

### Susceptibility assays

Overnight cultures of *F. novicida* or LVS strains were diluted to an OD_600_ of 1.0 and 100 μl of each were spread on mMH agar plates. Six mm filter disks (Bel-Art Scienceware, Lake Charles, LA) were then added to the center of each plate and 3 μl of the appropriate dilution of chemical agent was spotted on the disks. The following concentrations of chemical agents were used: 250 mM (*F. novicida*) and 25 mM (LVS) tert-butyl hydroperoxide, 150 mM cumene hydroperoxide, 3% H_2_O_2_, and 200 mg/ml sodium dodecyl sulfate (SDS). Plates were then grown overnight at 37°C and the zones of inhibition measured.

### Organic hydroperoxide degradation assay

Degradation of tert-butyl hydroperoxide was measured using a xylenol orange colorimetric assay based on previously described methods [59], [60], [75], [76]. Briefly, overnight cultures of *F. novicida* were subcultured to an OD_600_ of 0.01–0.03 and then incubated with shaking at 37°C. Once cultures reached mid-log phase (∼OD_600_ 1.0), they were diluted to OD_600_ 0.5 and a 2 ml sample of each culture or TSB alone were added to a 24 well plate. Tert-butyl hydroperoxide was then added to each sample to a final concentration of 300 µM, after which the plate was incubated with shaking at room temperature for 30 minutes. 20 µl samples were taken every five minutes from each well and immediately added to 80 µl 25 mM H_2_SO_4_. Once all samples were collected, 100 µl reaction buffer [200 µM xylenol orange (Alfa Aesar), 200 µM ammonium ferrous sulfate (Ricca Chemical, Arlington, TX), and 25 mM H_2_SO_4_ (Fisher Scientific) prepared in 9∶1 methanol to water solution] was added to each well and the OD_540_ measured. The concentration of tert-butyl hydroperoxide in each sample was calculated based on a standard curve.

### Statistical analysis

All macrophage replication, susceptibility, and qRT-PCR data were analyzed for significance using the unpaired Student's *t* test. The CI values from the mouse experiments were analyzed with the one-sample Student's *t* test and compared to 1, with the exception of the CI values comparing replication in wild-type versus gp91^phox-/-^ mice which were analyzed using the unpaired Student's *t* test. The single strain mouse infection data were analyzed for significance using the Mann-Whitney test.

## Results

### Intracellular replication screen

To further characterize the role in virulence of genes known to be required in animal infection models, we screened a library of corresponding transposon mutants for replication in RAW264.7 macrophages. We screened a total of 451 transposon mutants representing 224 genes and identified 53 of these genes to be required for *F. novicida* replication in RAW264.7 macrophages (Table 1). The screen results were validated by the identification of genes that have previously been reported to be required for intracellular replication, including genes encoded in the *Francisella* Pathogenicity Island (FPI) [8], [9], [11], [12], [77]–[80]. To the best of our knowledge, this study is the first to report the requirement of *Francisella*'s biotin biosynthetic genes and the *fsl*/*fig* siderophore biosynthetic genes for replication in mammalian cells [16], [53]–[55]. A more in-depth review of the genes identified in this screen is included in the Discussion. Interestingly, a large proportion of the genes identified to be required for intracellular replication by this screen encode proteins of unknown function. Two such proteins were chosen for further study: FTN_0096 and FTN_1133. FTN_0096 was selected because of the severe intracellular replication defect of the *FTN_0096* transposon mutant (Table 1). FTN_1133 was chosen because, although it is annotated as a hypothetical protein, we found that it has sequence similarity to Ohr, a protein involved in resistance to organic hydroperoxides which can induce oxidative stress, resistance to which is a critical virulence mechanism of *Francisella* species [17]–[21], [56]–[63].

**Table 1:** List of genes required for replication in RAW264.7 macrophages.

F. novicida locus	F. tularensis locus	Gene name	Gene description	Mammalian in vivo attenuation references	Mammalian in vitro attenuation references
FTN_0019	FTT1665	pyrB	aspartate carbamoyltransferase	[50]	[29], [32], [78], [89]
FTN_0020	FTT1664	carB	Carbamoyl-phosphate synthase large chain	[50], [112]	[29], [32], [78], [89]
FTN_0021	FTT1663	carA	Carbamoyl-phosphate synthase small chain	[50]	[29], [32], [78]
FTN_0035	FTT1648c	pyrF	Orotidine 5′-phosphate decarboxylase	[50]	[78]
FTN_0036	FTT1647c	pyrD	diyroorotate dehydrogenase	[50]	[78]
**FTN_0096**	**FTT1689c**	NA	**conserved hypothetical membrane protein**	[**50**]	[**78**]
FTN_0109	FTT1676	NA	hypothetical protein	[51], [108]	[78], [89], [108]
FTN_0177	FTT0203c	purH	bifunctional purine biosynthesis protein	[50], [112]	[29]
FTN_0178	FTT0204	purA	adenylosuccinate synthetase	[112], [113]	[29], [113]
FTN_0419	FTT0893	purM	Phosphoribosylaminoimidazol (AIR) synthetase	[50], [112]	
FTN_0422	FTT0896	purE	phosphoribosylaminoimidazole carboxylase,catalyic subunit	[50]	[114]
FTN_0430	FTT0904	lpnB	conserved hypothetical lipoprotein	[50], [52]	
FTN_0544	FTT0453c	NA	conserved hypothetical protein	[50]	
FTN_0546	FTT0455c	flmK	dolichyl-phosphate-mannose-protein mannosyltransferase family protein	[50], [115]	
FTN_0593	FTT0503c	sucD	succinyl-CoA synthetase, alpha subunit	[51]	[89], [114]
FTN_0594	FTT0504c	sucC	succinyl-CoA synthetase subunit beta	[51], [112]	[114]
FTN_0599	FTT0509c	NA	conserved hypothetical protein	[50]	
FTN_0812	FTT0934c	bioD	dethiobiotin synthetase	[51]	
FTN_0813	FTT0935c	bioC	biotin synthesis protein BioC	[51]	
FTN_0814	FTT0936c	bioF	8-amino-7-oxononanoate synthase	[50], [51]	
FTN_0815	FTT0937c	bioB	biotin synthase	[50]	
FTN_0816	FTT0938	bioA	Adenosylmethionine-8-amino-7-oxononanoate aminotransferase	[50]	
FTN_0818	FTT0941c	NA	lipase/esterase	[50], [51]	
FTN_0848	FTT0968c	NA	amino acid antiporter	[50], [51]	[78]
**FTN_1133**	**FTT1152**	**NA**	**protein of unknown function**	[**51]**, [52****]	
FTN_1240	FTT1221	NA	hypothetical protein	[51]	[78]
FTN_1254	FTT1236	NA	hypothetical protein	[50]	[29], [114]
FTN_1310	FTT1700; FTT1345	pdpB/icmF	conserved hypothetical protein	[50], [79], [112]	[78], [79]
FTN_1311	FTT1701; FTT1346	iglE	conserved hypothetical protein	[50]	[78]
FTN_1312	FTT1702; FTT1347	vgrG	conserved hypothetical protein	[9], [50], [52]	[9], [78]
FTN_1313	FTT1703; FTT1348	iglF	conserved hypothetical protein	[50]	[78]
FTN_1314	FTT1704; FTT1349	iglG	conserved hypothetical protein	[50]	[78]
FTN_1315	FTT1705; FTT1350	iglH	conserved hypothetical protein	[50]	[78]
FTN_1316	FTT1706; FTT1351	dotU	conserved hypothetical protein	[50]	[78]
FTN_1317	FTT1707; FTT1352	iglI	conserved hypothetical protein	[9], [50]	[9], [78]
FTN_1318	FTT1708; FTT1353	iglJ	conserved hypothetical protein	[50], [79]	[78], [79]
FTN_1321	FTT1711c; FTT1356c	iglD	intracellular growth locus, subunit D	[50], [52]	[12], [78], [80]
FTN_1322	FTT1712c; FTT1357c	iglC	intracellular growth locus, subunit C	[8], [50], [51], [116]	[8], [12], [77], [78], [116]
FTN_1323	FTT1713c; FTT1358c	iglB	intracellular growth locus, subunit B	[50], [51], [117]	[12], [78], [117]
FTN_1324	FTT1714c; FTT1359c	iglA	intracellular growth locus, subunit A	[50], [51]	[11], [12], [78]
FTN_1421	FTT1456c	wbtH	asparagine synthase	[50]	[89], [114]
FTN_1423	FTT1457c	wbtG	glycosyl transferase	[50]	
FTN_1427	FTT1461c	wbtD	galacturonosyl transferase	[50]	
FTN_1501	FTT1490	NA	Na+/H+ antiporter	[50]	[29], [91]
FTN_1586	FTT0129	NA	major facilitator superfamily sugar transporter		[29]
FTN_1608	FTT0107c	dsbB	disulfide bond formation protein	[29], [50], [91], [112]	[29], [78], [91]
FTN_1682	FTT0029c	figA/ fslA	conserved siderophore protein	[50], [51]	
FTN_1683	FTT0028c	figB/ fslB	conserved siderophore protein	[50]	
FTN_1684	FTT0027c	figC/fslC	diaminopimelate decarboxylase siderophore protein	[50]	
FTN_1699	FTT1720c	purL	phosphoribosylformylglycinamidine synthase	[50], [106], [112]	[91]
FTN_1700	FTT1721c	purF	amidophosphoribosyltransferase	[50], [106], [113]	[91], [113]
FTN_1715	FTT1736c	kdpD	two component sensor protein kdpD	[50], [118]	
FTN_1743	FTT1769c	clpB	ClpB protein	[50], [51], [92], [119]	[12], [78], [91], [92]

Strains highlighted in **bold** were chosen for further characterization in this study.

### Validation of Screen Results

**Figure 1:**
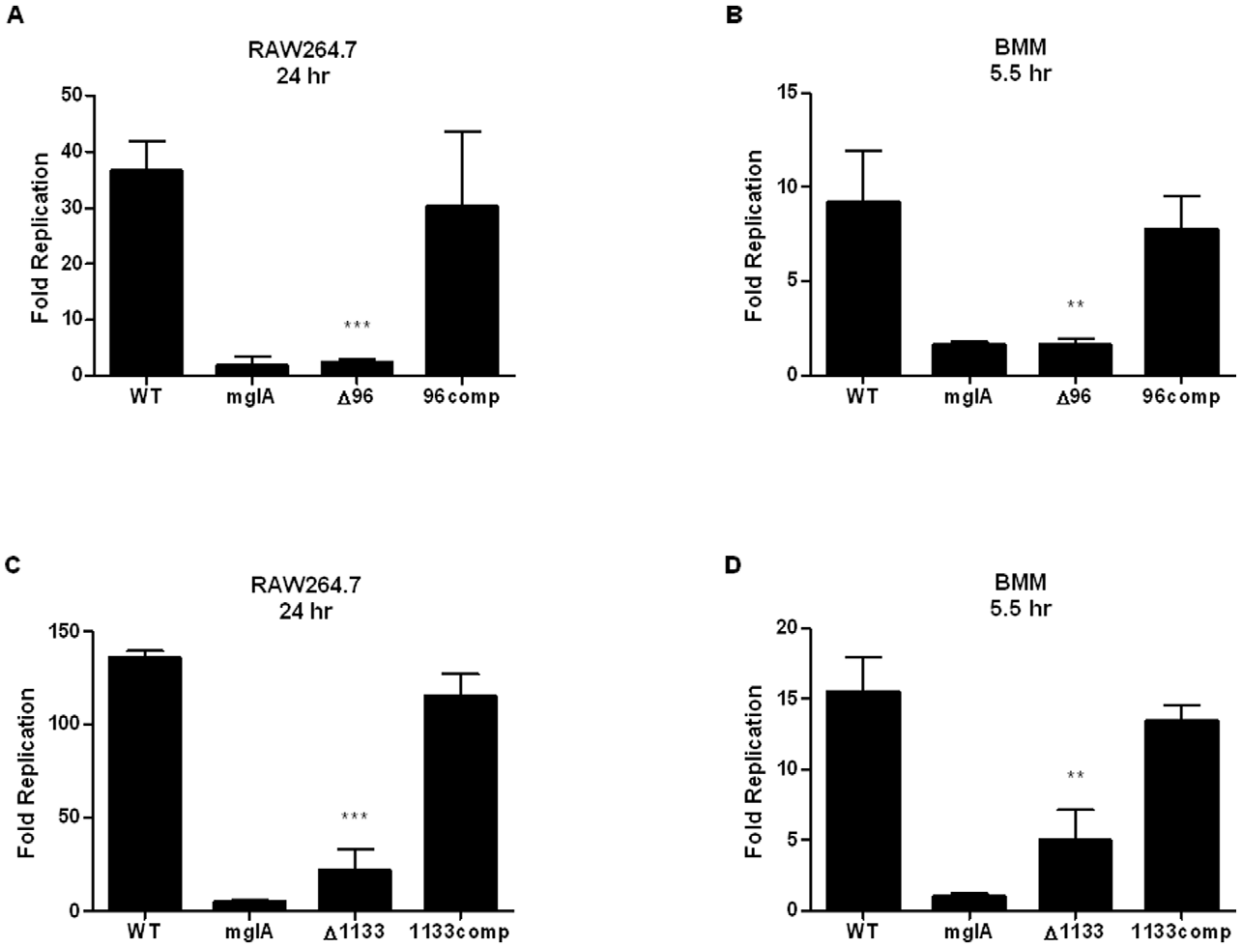
Validation of replication screen results. RAW264.7 macrophages (A and C) or primary murine bone marrow-derived macrophages (BMM) (B and D) were infected with a 20∶1 MOI of the indicated bacterial strains. Twenty-four (A and C) or 5.5 (B and D) hours post-infection, fold replication was determined for the *FTN_0096* (A and B) and *FTN_1133* (C and D) deletion mutants (Δ96 and Δ1133) and their respective complemented strains. Wild-type *F. novicida* and an *mglA* point mutant strain (GB2) were used as positive and negative replication controls, respectively. For each strain, bars represent the average fold replication and error bars represent the standard deviation (n = 3). Data shown is representative of at least three independent experiments. Asterisks indicate significance as compared to wild-type. (**) p<0.005, (***) p<0.0005.

Deletion mutants for *FTN_0096* and *FTN_1133* were generated using allelic replacement as previously described [70]. The mutants exhibited wild-type replication kinetics when grown in both tryptic soy broth (TSB) supplemented with cysteine (Fig. S1A) and Chamberlain's chemically defined minimal medium (Fig. S1B). To validate the phenotypes of the corresponding transposon mutants in our screen, the replication phenotype of each deletion mutant was determined in RAW264.7 macrophages. Twenty-four hours post-infection, the *FTN_0096* mutant was severely attenuated for replication compared to wild-type *F. novicida*, with a fold replication value similar to that of the replication-deficient control strain, GB2, which we will refer to as *mglA* (Fig. 1A). This strain harbors a point mutation in *mglA*, a gene known to be essential for intracellular replication [65]. The *FTN_1133* mutant displayed an approximate six-fold replication deficiency compared to wild-type (Fig. 1C). In order to ensure that the observed phenotypes resulted from deletion of the targeted gene and not unintended secondary site mutations, we generated complemented strains of each mutant in which the deleted gene was replaced. All of the complemented strains displayed wild-type levels of replication (Fig. 1A, C). These data further validate our screen and demonstrate the requirement of *FTN_0096* and *FTN_1133* for *F. novicida* replication in RAW264.7 macrophages.

To verify that the replication defects of these *F. novicida* mutants were not specific to RAW264.7 cells, we next infected primary murine bone marrow-derived macrophages (BMM) and measured replication levels. Compared to the wild-type strain, the *FTN_0096* mutant again displayed a severe replication deficiency similar to the *mglA* mutant, and the *FTN_1133* mutant had an approximate three-fold replication defect (Fig. 1B, D). Replication was restored to the wild-type level in the complemented strains (Fig. 1B, D). We measured bacterial replication at 5.5 hrs post-infection, before any macrophage cell death occurred, to ensure that the attenuated phenotypes of the mutants were not a consequence of the cell death response. RAW264.7 macrophages are known to be deficient in ASC/caspase-1 inflammasome-mediated cell death [81], an inflammatory host cell death pathway known to be triggered by *F. novicida* infection [82], explaining why we could measure bacterial replication at later time points in these cells. Taken together, these results demonstrate that in both of the macrophage cell types tested, *FTN_0096* and *FTN_1133* play a role in replication and that the deletion mutants lacking these genes displayed intracellular replication deficiencies similar to those predicted by our screen.

### Deletion mutants are attenuated *in vivo*

**Figure 2:**
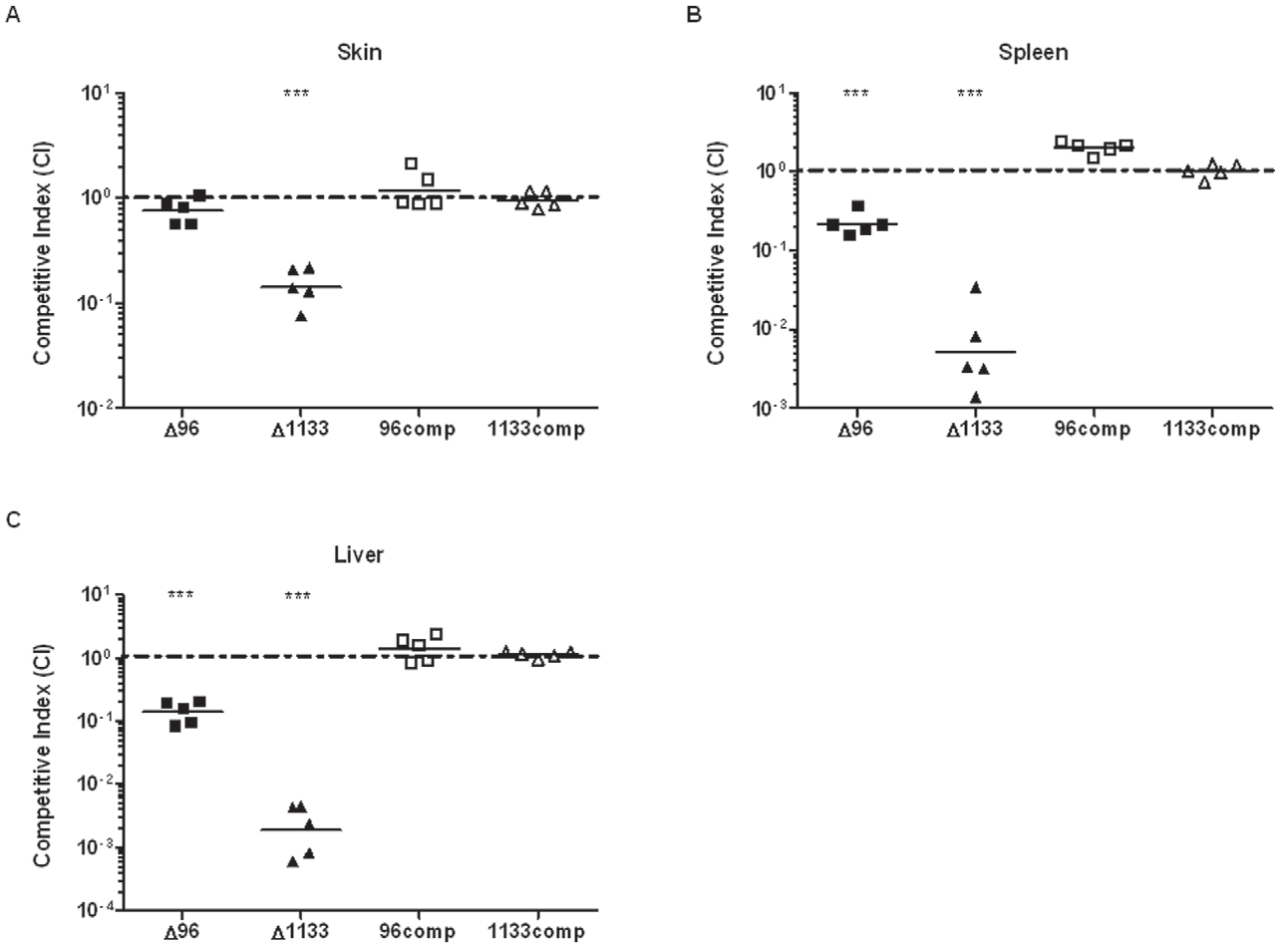
*FTN_0096* and *FTN_1133* deletion mutants are attenuated for virulence *in vivo*. Mice were subcutaneously infected with a 1∶1 mixture of wild-type *F. novicida* and either Δ96 or Δ1133 (10^5^ CFU each) or respective complemented strains. Forty-eight hours after infection, organs were harvested, CFU enumerated, and the competitive index (CI) calculated for the skin at the site of infection (A), spleen (B), and liver (C). CI  =  (CFU mutant output/CFU WT output)/(CFU mutant input/CFU WT input). Bars represent the geometric mean CI values from each group of mice (n = 5). CI values below 1 (dashed line) indicate attenuation of the mutant strain. Data shown is representative of two independent experiments. Asterisks indicate significance as compared to a CI value of 1. (***) p<0.0005.

To test whether the macrophage replication defects correlated with *in vivo* attenuation levels, competition experiments were performed in mice. Briefly, mice were infected with a 1∶1 ratio of wild-type *F. novicida* and each mutant strain. Forty-eight hours post-infection, mouse organs were harvested, homogenized, and plated for enumeration of wild-type and mutant CFU. The number of *FTN_0096* deletion mutant CFU in the spleen and liver was one log below that of wild-type (Fig. 2B, C), although no attenuation was observed in the skin (Fig. 2A). The *FTN_1133* mutant had a one log attenuation in the skin, roughly two log attenuation in the spleen, and nearly three log attenuation in the liver (Fig. 2). Both mutant phenotypes were restored to wild-type levels in the complemented strains (Fig. 2). Taken together, these results demonstrate that FTN_0096 and FTN_1133 are involved in *F. novicida* pathogenesis *in vivo*, though FTN_1133 appears to have a more significant role in virulence in mice.

### *FTN_1133* is required for virulence in single infections

**Figure 3:**
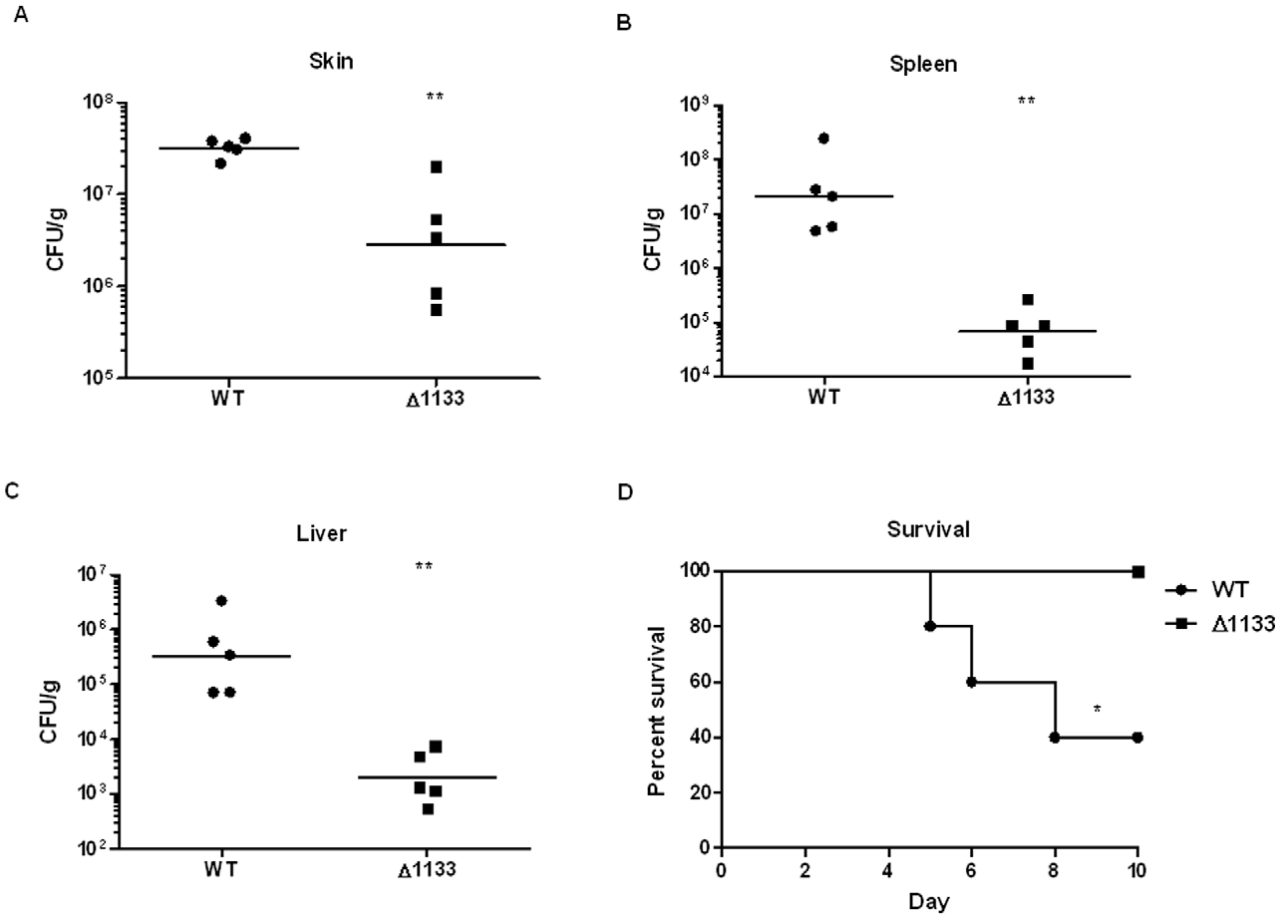
FTN_1133 is required for *F. novicida* pathogenesis in single infections. Mice were subcutaneously infected with 2×10^5^ CFU of either wild-type *F. novicida* (WT) or the *FTN_1133* mutant (Δ1133). Forty-eight hours after infection, organs were harvested and plated and CFU were enumerated 24 hours later for the skin at the site of infection (A), spleen (B), and liver (C). To test survival, mice were infected as described above and then sacrificed upon display of moribundity (D). Bars represent the geometric mean of each group of mice (n = 5). Data shown is representative of two independent experiments. Asterisks indicate significance. (**) p<0.005, (*)  =  P<0.05.

Since the *FTN_1133* mutant was the most severely attenuated *in vivo*, we chose this gene for further characterization. To ensure that the attenuation of this mutant was not only observed when in competition with wild-type bacteria, we performed single infection experiments. We observed that the *FTN_1133* mutant was attenuated in each organ to a similar degree as in competition experiments (Fig. 3A–C), confirming that FTN_1133 is required for full virulence of *F. novicida*. We further verified this result by monitoring the survival of these mice. By day 10 post-infection, only 40% of mice infected with wild-type bacteria survived while 100% of those infected with the *FTN_1133* deletion mutant survived (Fig. 3D).

### *FTN_1133* is expressed during infection of macrophages and mice

**Figure 4:**
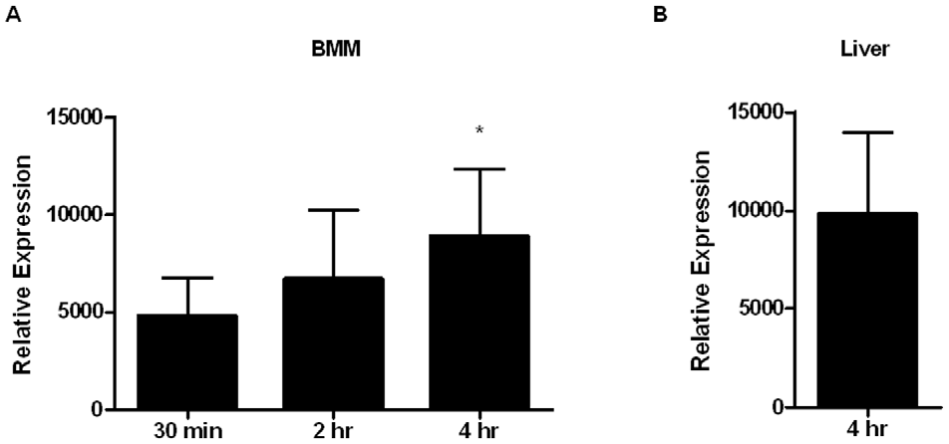
*FTN_1133* is expressed during infection of macrophages and mice. Relative expression of *FTN_1133* was determined in wild-type *F. novicida*-infected BMM (A) at 30 minutes, 1 hour, and 4 hours post-infection with an MOI of 20∶1 and (B) in the livers of intraperitoneally-infected mice at 4 hours. Relative expression of *FTN_1133* transcript was calculated by comparison with the expression levels of the DNA helicase *uvrD* (*FTN_1594*). Data shown is representative of two independent experiments. Bars represent the standard deviation from each set of samples (n = 5). Asterisk indicates significance compared to 30 minutes. (*) p<0.05.

Given its importance in *F. novicida* replication and survival in macrophages and mice, we tested whether *FTN_1133* was transcribed during infection. Levels of *FTN_1133* transcript from samples of wild-type *F. novicida*-infected BMM at 30 minutes, 2 hours, and 4 hours post-infection were determined using quantitative real-time RT-PCR (qRT-PCR) (Fig. 4A). The expression of *FTN_1133* was induced during macrophage infection, as indicated by a moderate but significant increase in expression between 30 minutes and 4 hours (Fig. 4A). We also observed a similar level of expression in livers of infected mice 4 hours after intraperitoneal infection (Fig. 4B). Taken together and consistent with its role in pathogenesis, these results show that *FTN_1133* is expressed during infection of both macrophages and mice.

### FTN_1133 is involved in resistance to organic hydroperoxides

**Figure 5:**
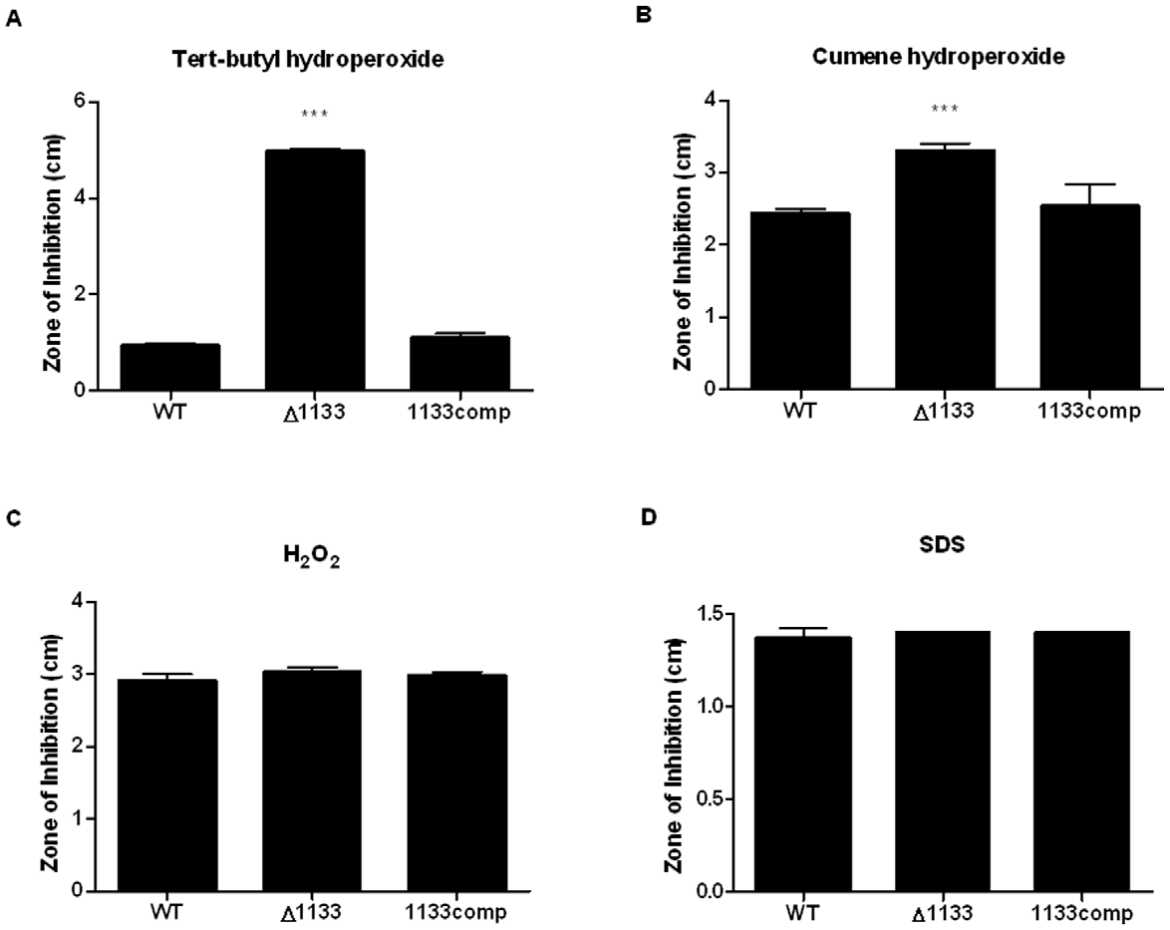
FTN_1133 confers resistance to organic hydroperoxides. Two hundred and fifty mM tert-butyl hydroperoxide (A), 150 mM cumene hydroperoxide (B), 3% hydrogen peroxide (C), and 200 mg/ml SDS (D) were spotted on filter disks placed on lawns of wild-type, *FTN_1133* mutant (Δ1133), and the complemented strain and the zones of inhibition for each were measured. In each graph, bars represent the mean and error bars represent the standard deviation (n = 3). Data shown is representative of at least three independent experiments. Asterisks indicate significance as compared to wild-type. (***) p<0.0005.

Bioinformatic analysis revealed that FTN_1133 has sequence similarity to an organic hydroperoxide resistance protein (Ohr) from *Bacillus megaterium*. Specifically, the C-terminal half of Ohr has significant similarity to residues 18–86 of FTN_1133 (Fig. S2). Based on these findings, we tested whether FTN_1133 is involved in resistance to organic hydroperoxides in *F. novicida*. We quantified bacterial sensitivity by measuring zones of inhibition upon exposure to organic hydroperoxides via the disk diffusion method. The *FTN_1133* mutant showed increased sensitivity to cumene and tert-butyl hydroperoxides compared to the wild-type and complemented strains (Fig. 5A, B). Since Ohr is often required for resistance to organic hydroperoxides but not inorganic hydroperoxides [59], [60], [62], [63], [83], we grew the bacterial strains in the presence of inorganic hydrogen peroxide and found that the wild-type, mutant, and complemented strains showed equal levels of sensitivity, indicating that FTN_1133 is indeed not required for resistance to an inorganic hydroperoxide (Fig. 5C). Finally, to further demonstrate the specificity of FTN_1133 for organic hydroperoxides and rule out a general sensitivity to stresses, we found that the wild-type, mutant and complemented strains were equally susceptible to SDS, a membrane-damaging detergent (Fig. 5D). Taken together, these data demonstrate that FTN_1133, similar to most Ohr proteins, is required for resistance to organic but not inorganic hydroperoxides.

### FTN_1133 is required for degradation of an organic hydroperoxide

**Figure 6:**
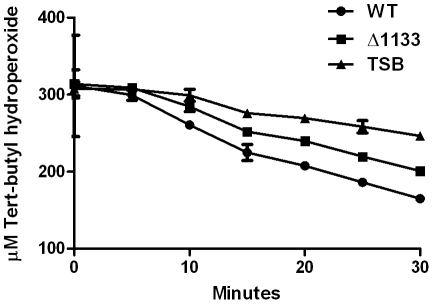
FTN_1133 is required for degradation of an organic hydroperoxide. Three hundred μM tert-butyl hydroperoxide was added to cultures of wild-type *F. novicida* (circles), the *FTN_1133* deletion mutant (Δ1133, squares), or TSB media alone (triangles) and its degradation was measured over time. Data points represent the mean and error bars represent the standard deviation (n = 3). Data shown is representative of at least three independent experiments.

To determine if FTN_1133 is important not only for resistance to organic hydroperoxides, but also detoxification of these chemicals, we added tert-butyl hydroperoxide to cultures of wild-type *F. novicida* or the *FTN_1133* deletion mutant and measured its concentration over time. Between 15 minutes and 30 minutes, the *FTN_1133* deletion mutant degraded approximately 50% less tert-butyl hydroperoxide than wild-type bacteria (Fig. 6). These data indicate that FTN_1133 is involved in degradation of an organic hydroperoxide.

### FTN_1133 is required for resistance to the action of the NADPH oxidase

**Figure 7:**
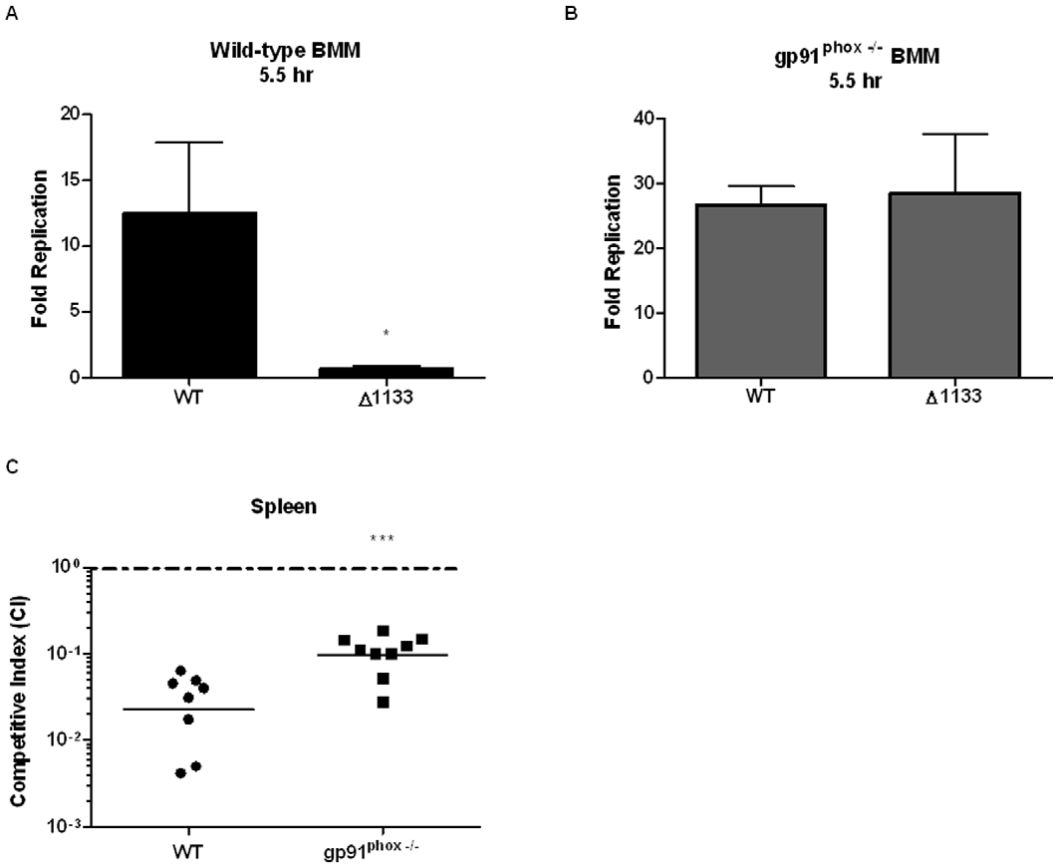
FTN_1133 is required for resistance to the action of the NADPH oxidase. Wild-type (A) or gp91^phox-/-^ (B) bone marrow-derived macrophages were infected with a 20∶1 MOI of the indicated bacterial strains. (A, B) Five and a half hours post-infection, fold replication (CFU at 5.5 hr/ CFU at 30 minutes) was determined for wild-type *F. novicida* and the *FTN_1133* deletion mutant (Δ1133). For each strain, bars represent the average fold replication and error bars represent the standard deviation (n = 3). Data shown are representative of three independent experiments. (C) Wild-type and gp91^phox-/-^ mice were subcutaneously infected with a 1∶1 mixture of wild-type *F. novicida* and Δ1133 (10^5^ CFU each). Forty-eight hours after infection, organs were harvested, CFU enumerated, and the competitive index (CI) calculated. Data shown include two independent experiments. Asterisks indicate significance as compared to wild-type *F. novicida*. (*) p<0.05, (***) p<0.0005.

To test if the sensitivity of the *FTN_1133* mutant to oxidative stress was the cause of its replication defect in macrophages, we infected BMM from both wild-type and gp91^phox-/-^ mice with wild-type *F. novicida* and the *FTN_1133* deletion mutant. gp91 is a subunit of the NADPH oxidase and is required for the generation of reactive oxygen species and the oxidative stress induced by this enzyme. While the *FTN_1133* mutant was attenuated for replication in wild-type BMM (Fig. 7A, and similar to Fig. 1), it replicated to the same levels as wild-type *F. novicida* in the gp91^phox-/-^ BMM (Fig. 7B). In order to determine whether a similar phenotype is observed *in vivo*, we infected wild-type and gp91^phox-/-^ mice with either wild-type *F. novicida* or the *FTN_1133* deletion mutant. Indeed, the two log attenuation of the *FTN_1133* deletion mutant in wild-type mice was rescued by one log in gp91^phox-/-^ mice (Fig. 7C). Together, these data indicate that FTN_1133 is required to resist the oxidative stress generated by the NADPH oxidase and makes an important contribution to *F. novicida*'s intracellular and *in vivo* pathogenesis.

### The *FTN_1133* ortholog, *FTL_0803*, confers resistance to an organic hydroperoxide

**Figure 8:**
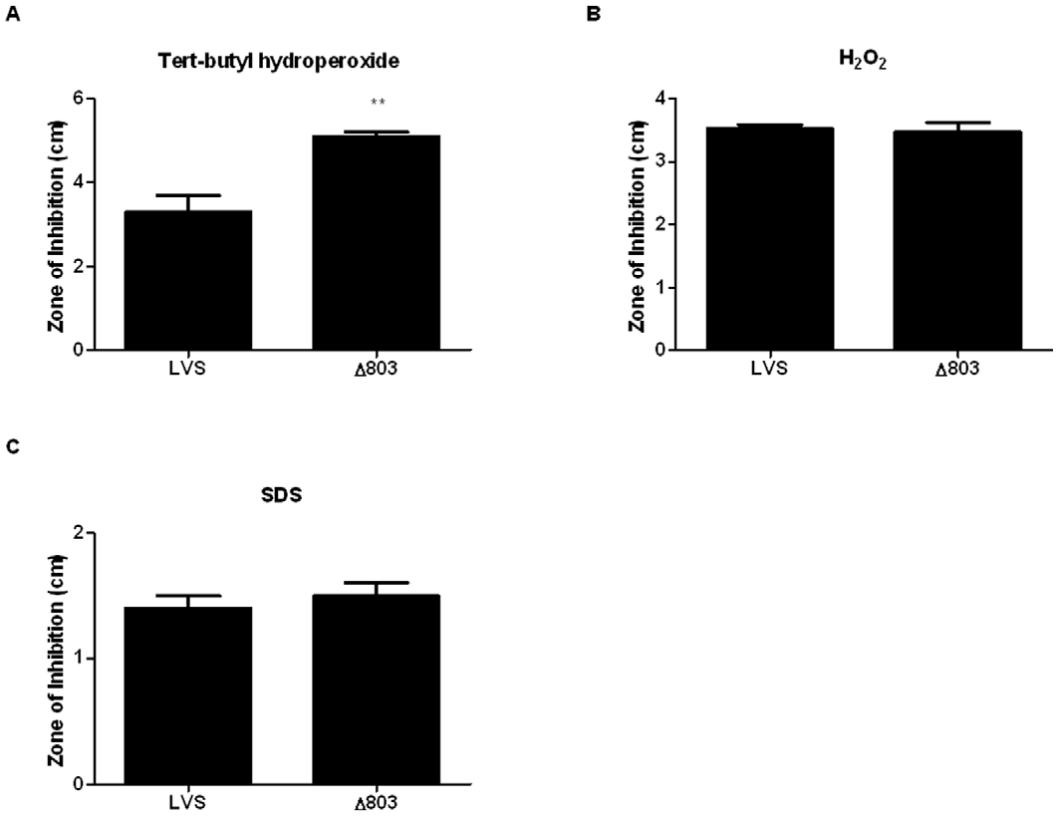
The *FTN_1133* ortholog, *FTL_0803*, confers resistance to tert-butyl hydroperoxide. Twenty-five mM tert-butyl hydroperoxide (A), 3% hydrogen peroxide (B), and 200 mg/ml SDS (C) were spotted on filter disks placed on lawns of wild-type LVS or the *FTL_0803* mutant (Δ803), incubated overnight, and then the zones of inhibition for each were measured. In each experiment, bars represent the mean and error bars represent the standard deviation (n = 3). Data shown is representative of at least three independent experiments. Asterisks indicate significance as compared to wild-type. (**) p<0.005.

In order to determine whether *FTN_1133* is also involved in organic hydroperoxide resistance in other *Francisella* species, we constructed an *F. holarctica* LVS deletion mutant lacking the *FTN_1133* ortholog, *FTL_0803*. FTN_1133 and FTL_0803 share 98% amino acid identity. To test the requirement of FTL_0803 for LVS resistance to organic hydroperoxides, we performed disk diffusion assays on wild-type LVS and the *FTL_0803* deletion mutant. As with FTN_1133, we found that FTL_0803 is required for wild-type resistance to the organic hydroperoxide tert-butyl hydroperoxide (Fig. 8A), but not H_2_O_2_ (Fig. 8B) or SDS (Fig. 8C).

### FTL_0803 is required for *F. holarctica* LVS virulence and resistance to the action of the NADPH oxidase

**Figure 9:**
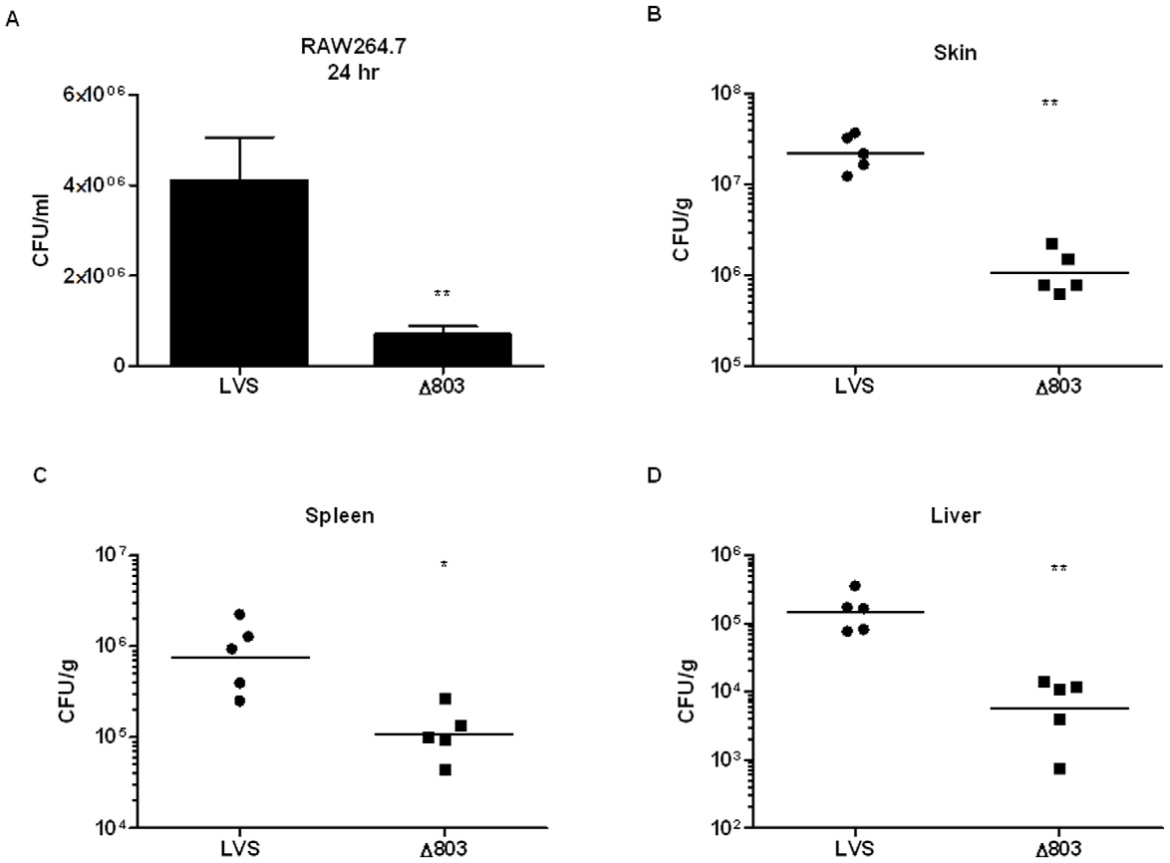
FTL_0803 is important for LVS replication in macrophages and mice. (A) RAW264.7 macrophages were infected with a 20∶1 MOI of wild-type LVS and the *FTL_0803* mutant (Δ803). Twenty-four hours post-infection, intracellular CFUs were determined for both strains. Bars represent the mean and error bars represent the standard deviation (n = 3). (B–D) Mice were subcutaneously infected with 2×10^5^ CFU of either wild-type *F. holarctica* LVS (WT) or the *FTL_0803* mutant (Δ803). Seventy-two hours after infection, organs were harvested, plated, and CFU were enumerated 48 hours later for the skin at the site of infection (B), spleen (C), and liver (D). Bars represent the geometric mean from each group of mice (n = 5). Data shown is representative of two independent experiments. Asterisks indicate significance as compared to wild-type. (*) p<0.05, (**) p<0.005.

**Figure 10:**
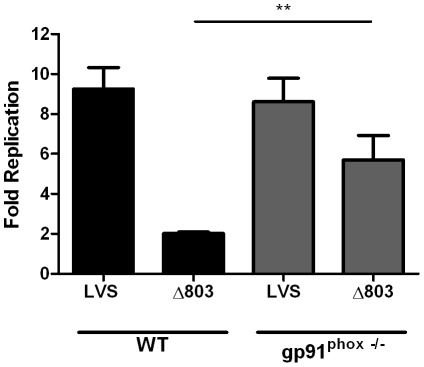
FTL_0803 contributes to resistance against the action of the NADPH oxidase. Bone marrow-derived macrophages (BMM) were infected with a 20∶1 MOI of wild-type LVS or the *FTL_0803* mutant (Δ803). Twenty-four hours post-infection, fold replication was determined for both strains. Bars represent the mean and error bars represent the standard deviation (n = 3). Data shown is representative of two independent experiments. Asterisks indicate significance as compared to wild-type. (**) p<0.005.

Next we investigated the importance of FTL_0803 in LVS pathogenesis by determining the replication phenotype of the deletion mutant both in macrophages and mice. First, RAW264.7 macrophages were infected with wild-type LVS and the *FTL_0803* mutant, and bacterial levels were measured at 24 hours post-infection. Similar to the phenotype of the *FTN_1133* mutant, the *FTL_0803* mutant exhibited a five-fold intracellular replication defect (Fig. 9A). Mouse infection experiments revealed that the *FTL_0803* deletion mutant was present at one log lower levels than LVS in the organs tested (Fig. 9B–D). Finally, to determine if the replication deficiency of this mutant in macrophages is also rescued in the absence of a functional NADPH oxidase, we infected both wild-type BMM and gp91^phox-/-^ BMM with either LVS or the *FTL_0803* deletion mutant. The five-fold replication deficiency of the mutant in wild-type BMM was largely rescued in gp91^phox-/-^ BMM (Fig. 10). These data demonstrate that the importance of FTN_1133/FTL_0803 in resisting oxidative stress, promoting intracellular replication, and contributing to *in vivo* virulence is conserved in multiple *Francisella* species.

## Discussion

Recently, *in vivo* screens have identified many genes required for *Francisella* virulence, though the functions of these genes and an understanding of the stage of infection at which they contribute to virulence are largely unknown [50], [51]. In an effort to further characterize these virulence determinants and to begin to answer questions that remain regarding *Francisella*'s intracellular lifecycle, we performed an intracellular replication screen using transposon insertion mutants of genes that were identified in the two mammalian *in vivo* genome-wide screens that were published at the time of this study: one using *F. novicida* transposon mutants [50] and one using transposon mutants of the *Francisella* live vaccine strain (LVS) [51]. Recently, Kraemer et al. published another mammalian *in vivo* genome-wide screen, the results of which were published after our screen was performed and therefore genes specifically identified in this study were not included [52].

Of the 224 genes screened, 53 were shown to be required for replication in murine macrophages. The 171 genes that were not identified in this screen may be required for replication in other cell types known to be infected by *Francisella*, such as neutrophils, hepatocytes, alveolar epithelial cells, or fibroblasts [29]–[32], [84], [85]. Alternatively, these genes may be required for processes other than intracellular replication such as immune evasion or systemic dissemination. To the best of our knowledge, the intracellular replication data from mutants for 140 of the 224 genes that were represented in this screen have not been previously reported in mammalian cells. One gene, *feoB* (*FTN_0066*), was included in the screen but was excluded from the results because of a growth defect on cysteine-enriched tryptic soy agar (TSA), despite no apparent growth defect in cysteine-enriched tryptic soy broth (TSB) (data not shown).

The results of our screen were validated by the identification of genes that have previously been shown to be required for replication in macrophages, including most of the *Francisella* pathogenicity island (FPI) genes [8], [9], [11], [12], [77]–[80], [86]. Of the 18 genes in the *F. novicida* FPI, mutants representing 13 genes were identified as attenuated for replication in macrophages. Among the FPI genes not identified in this screen (*pdpA*, *pdpC*, *pdpD*, *pdpE*, and *anmK*), *pdpD* has previously been reported to be unnecessary for replication in mouse macrophages [87]. Both *pdpC* and *pdpD* were also shown to be unnecessary for replication in an arthropod cell line [88] and *pdpC*, *pdpD*, *pdpE*, and *anmK* were all reported to have no role in virulence in a live arthropod model [78]. While *pdpA* has been shown to be required for intracellular replication within mammalian cells [10], [78], [86], it was not identified in this screen. One of the three *pdpA* transposon mutants we tested displayed a deficiency in intracellular replication, but it was just below the cut-off value used in this screen (Table S1). This may be due to retention of some protein function in the *pdpA* mutant strains used in this study, a potential problem when using transposon mutants as compared to deletion mutants.

In addition to the identification of FPI genes, this screen was further validated by the identification of other genes that have previously been shown to be required for intracellular replication, including *pyrB* [29], [32], [78], *carA* [29], [32], [78], *carB* [29], [32], [78], [89], *purM* [90] and *clpB* [12], [78], [91], [92]. Genes that have previously been shown to play no role in intracellular replication and were also shown to be unnecessary for replication in our screen include *FTN_0757* and *FTN_0720*, verifying the selectivity of our screen [50]. Though an *htpG* deletion mutant has previously been reported to be attenuated for replication in BMM macrophages [50], and one of the two transposon mutants with insertions in this gene displayed an approximate two-fold replication attenuation in this screen, the level of attenuation was not sufficient for our cut-off.

Our screen led to numerous novel insights into *Francisella*'s genetic requirements for replication within host cells. To the best of our knowledge, this is the first study to implicate a requirement of biotin for *Francisella's* replication in mammalian cells since each of the biotin synthetic genes, *bioA*, *bioB*, *bioC*, *bioD*, and *bioF* (*FTN_0812*-*FTN_0816*) was identified. Biotin is critical for various metabolic pathways and biotin biosynthetic genes are required for *Mycobacterium tuberculosis* intracellular replication and pathogenesis [93]–[95]. *bioF* was also recently reported to be important for *Francisella* replication in an arthropod cell line [96]. In addition, we report here for the first time *Francisella*'s requirement for replication in mammalian cells of at least three of the six siderophore biosynthetic genes: *fslA*, *fslB*, and *fslC*, also known as *figA*, *figB*, and *figC* (*FTN_1682*-*FTN_1684*), confirming that this iron acquisition system is essential for efficient replication within mammalian cells. Pathogens generally encounter iron-limiting conditions within the host, and thus iron acquisition proteins are critical virulence factors in numerous pathogens [97], [98]. *fslA* and *fslB* were also recently reported to be important for replication in an arthropod cell line [96]. The other three genes from this group (*FTN_1685*-*FTN_1687*) were not included in our screen.

Of the 53 genes identified to be required for replication in this screen, 19 are annotated as hypothetical, indicating that *F. novicida* encodes novel genes that are required both for virulence *in vivo* and intracellular replication. To study the role of some of these hypothetical proteins during infection as well as further validate the screen results, we chose two genes to study: *FTN_0096* and *FTN_1133*. FTN_0096 is a member of the DUF1275 superfamily of proteins, which has members in other human pathogens such as *Vibrio* spp, *Acinetobacter* spp, *Neisseria* spp, and *Burkholderia* spp. Analysis with the Simple Modular Architecture Research Tool (SMART) identified 7 potential transmembrane domains as well as a putative signal peptide, indicating that FTN_0096 is likely a membrane protein (http://smart.embl-heidelberg.de/). Interestingly, bioinformatic analyses revealed that FTN_1133 was found only in *Francisella* species but had significant similarity to the C-terminal domain of an organic hydroperoxide resistance protein, Ohr, from *Bacillus megaterium*.

We show that both *FTN_0096* and *FTN_1133* are required for replication in macrophages and virulence in mice. FTN_0096 was absolutely required for replication in both RAW264.7 macrophages and BMM (Fig. 1A, B). In addition, *FTN_0096* was recently reported to be important for replication in an arthropod *in vivo* model as well as murine macrophage-like J774 cells [78]. The *FTN_0096* deletion mutant displayed only a moderate one log attenuation in the spleen and liver of mice, despite having a severe intracellular replication defect (Fig. 2). The disparity in this mutant's *in vivo* versus *in vitro* phenotypes could be due to an ability of bacteria lacking *FTN_0096* to replicate efficiently in other cells types, particularly non-immune cells that have fewer defenses against intracellular infection. Indeed, Horzempa et al. recently showed that a uracil synthesis mutant unable to replicate in macrophages displayed a virulent phenotype in mice attributable to that mutant's ability to replicate in non-phagocytic host cells [99]. Conversely, the *FTN_1133* mutant was moderately attenuated for intracellular replication (3-6 fold) in macrophages (Fig. 1), displayed a marked two to three log attenuation in the spleen and liver following mouse infections (Fig. 2, 3), and did not cause lethal infection in mice (Fig. 3D). Furthermore, we showed that *FTN_1133* was expressed during infection of both macrophages and mice (Fig. 4).

Bioinformatic analyses revealed that FTN_1133, a 127 amino acid protein, has significant similarity to the C-terminal domain of an organic hydroperoxide resistance protein (Ohr) from *Bacillus megaterium* (Fig. S2). The C-terminus of Ohr proteins has been shown to be important for enzymatic function in bacteria such as *Xylella fastidiosa* and *Pseudomonas aeruginosa* [83], [100]–[102]. Originally identified in *Xanthomonas campestris*, Ohr is a 139 amino acid protein which has homologs in several bacterial species including *Bacillus subtilis*, *Pseudomonas aeruginosa*, and *Acinetobacter baumannii* [56], [57], [59], [61]. Ohr is thought to be a hydroperoxide reductase that converts organic hydroperoxides into less toxic metabolites [101]. This protein contributes to resistance to reactive oxygen species (ROS)-induced damage by degrading the highly toxic organic hydroperoxides that are created during lipid peroxidation when oxygen radicals react with the unsaturated and polyunsaturated lipids of cell membranes [37]–[40], [100].

Interestingly, Ohr proteins are usually only involved in resistance to organic hydroperoxides, such as tert-butyl hydroperoxide and cumene hydroperoxide, but not inorganic hydroperoxides, such as hydrogen peroxide [59], [60], [62], [63], [83]. Our disk diffusion analysis indicates that FTN_1133, like many Ohr proteins, is required for resistance to organic hydroperoxides but not hydrogen peroxide (Fig. 5). Also similar to Ohr, we demonstrate that FTN_1133 is required for degradation of tert-butyl hydroperoxide (Fig. 6) [59], [60], [75], [76]. The *FTN_1133* deletion mutant displays a moderate amount of degradation compared to the wild-type strain, which indicates that *Francisella* may encode another system involved in degradation of these chemicals. Potential candidates for this function include *Francisella*'s uncharacterized AhpC homologs. Most proteins involved in oxidative stress resistance specifically detoxify inorganic oxygen species, such as oxide radicals (superoxide dismutases) or hydrogen peroxide (catalase). However, AhpC has been shown to be important for resistance to both inorganic and organic hydroperoxides in other bacterial pathogens such as *P. aeruginosa*, *Brucella abortus*, and *Salmonella typhimurium* [103]–[105]. Consistent with a role in virulence, Kadzhaev et al. demonstrated that an *F. tularensis ahpC* transposon mutant showed a marked increase in time to death in a low dose challenge in mice [106].

While expression of *ohr* genes is often increased in response to organic hydroperoxides, several *ohr* genes have been identified that are not induced in this manner [57], [62], [64]. Quantitative real-time PCR analysis of *FTN_1133* expression revealed that while the gene is induced during infection (Fig. 4), this gene was not significantly induced in broth in our hands in response to organic hydroperoxides (data not shown).

To the best of our knowledge, *ohr* mutants have not previously been characterized in host cells or animal models, though *ohr* has been shown to be co-expressed with other virulence factors during *Actinobacillus pleuropneumoniae* infection of pigs [60]. Further suggestive of a role in pathogenesis, *ohr* is sometimes encoded on mobile genetic elements, such as a genomic island in *Actinobacillus pleuropneumoniae* [107] and a plasmid in pathogenic *Acinetobacter baumannii* [61]. Our *in vitro* screen and previous *in vivo* screens [51], [52] identified *FTN_1133* as important for virulence. We hypothesized that increased sensitivity of the *FTN_1133* mutant to oxidative stress may explain its intracellular and *in vivo* replication defect. Indeed, we observed restoration of wild-type levels of intracellular replication for the *FTN_1133* deletion mutant in BMM from mice lacking gp91^phox-/-^ (Fig. 7B), an essential subunit of NADPH oxidase that is required for the generation of superoxide radicals by this enzyme complex. In addition, the *in vivo* attenuation of the *FTN_1133* mutant was significantly rescued in gp91^phox-/-^ mice (Fig. 7C). Though the mutant was not fully complemented in these mice as it was in the gp91^phox-/-^ BMM, this is not surprising since there are alternate ROS generating pathways that do not exist in macrophages but are present during *in vivo* infection. For example, myeloperoxidase is present in neutrophils but not macrophages and can initiate lipid peroxidation and organic hydroperoxide generation [41]. Furthermore, we demonstrate that the importance of FTN_1133 in resistance to oxidative stress is conserved in multiple *Francisella* species by showing the requirement of *FTL_0803*, the *F. holarctica* LVS *FTN_1133* ortholog, for resistance to organic hydroperoxides (Fig. 8) and wild-type replication both in macrophages and mice ( Fig. 9). Finally, we show that the *in vitro* attenuation of the *FTL_0803* mutant was largely rescued in macrophages deficient in oxidative burst (Fig. 10). The molecular bases for the attenuation of the LVS strain have yet to be fully characterized and as such, there may be underlying deficiencies that explain the incomplete complementation of the *FTL_0803* mutant strain in gp91^phox-/-^ BMM [26].

Our data demonstrating a role for FTN_1133 in resistance to oxidative stress and *Francisella* virulence are consistent with the fact that many oxidative stress resistance genes are transcribed during *Francisella* infection of macrophages [108] and many of these have been identified in screens as being required for replication in macrophages [78], [89] and virulence in arthropods [20], [78], [96]. *Francisella* species have been shown to use an array of genes to suppress activation of the NADPH oxidase [32], [35], [36], [45], [109]. *Francisella* species also use numerous genes to detoxify reactive oxygen compounds and thereby resist oxidative stress. Specifically, the catalase KatG [48], [49], [51], [89] and the superoxide dismutases SodB and SodC [18], [49], [110] have all been shown to be essential for survival of *Francisella* species *in vivo*. Also, a novel oxidative stress resistance gene, MoxR, was recently described in LVS [21]. The data presented here identify FTN_1133 as a novel *Francisella* oxidative stress resistance protein, specific for stress induced by organic hydroperoxides.

In this report, we demonstrate for the first time the importance of an Ohr-like protein in virulence during *in vitro* and *in vivo* infections as well as its specific role in resistance to oxidative stress both in macrophages and in mice. Furthermore, we show that the importance of this protein for pathogenesis is conserved in multiple *Francisella* species. Taken together, the results of this screen highlight the requirement of numerous *F. novicida* virulence determinants for intracellular replication. The critical importance of resisting oxidative stress suggests that Ohr-like proteins, including FTN_1133, may represent attractive drug targets [111]. In this way, continued characterization of FTN_1133 and other novel proteins and mechanisms used by *Francisella* could contribute to the development of new therapeutics and vaccines against this potential bio-threat.

## Supporting Information

Figure S1 **Selected deletion mutants of genes identified in the replication screen display wild-type growth in rich media and in defined minimal media.** Bacterial growth at 37°C in (A) cysteine-enriched tryptic soy broth and (B) Chamberlain's minimal defined media is shown for wild-type *F. novicida* (circles), *FTN_0096* (Δ96, squares), and *FTN_1133* (Δ1133 triangles). Data shown is representative of at least three independent experiments.
(TIF)

Figure S2 **FTN_1133 has similarity to the organic hydroperoxide resistance protein Ohr.** The sequences for *F. novicida* FTN_1133 (a.a. 18 – 86) and *Bacillus megaterium* Ohr (a.a. 71 – 139) were aligned using CLUSTALW (http://www.ebi.ac.uk/Tools/msa/clustalw2/). Identical residues are highlighted in red and similar residues are highlighted in blue. The sequences have 28.6% identity and 42.9% similarity.
(TIF)

Table S1 **Full list of transposon mutant replication phenotypes in RAW264.7 macrophages.** Strains highlighted in **bold** were attenuated for intracellular replication. Each transposon mutant was screened 2–3 times and each time the fold replication of the mutant was compared to the fold replication of wild-type (fold Mut/ fold WT). AVG Mut/WT is the average of these ratios from all experiments for each mutant. Strain names, plate number, and well location are as annotated in the two-allele transposon mutant library from Gallagher, et al.
(DOC)

Table S2 **Primers used in this study.**
(DOC)
