# Supplementary figures and images for: Macrophage Replication Screen Identifies a Novel Francisella Hydroperoxide Resistance Protein Involved in Virulence
Source: PLoS One. 2011 Sep 6;6(9):e24201. doi: 10.1371/journal.pone.0024201 (PMC3167825; doi:10.1371/journal.pone.0024201)

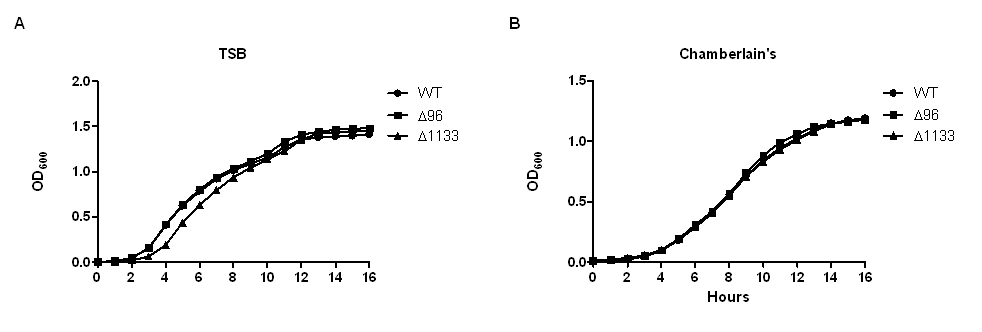

Supplement: Figure S1 — Selected deletion mutants of genes identified in the replication screen display wild-type growth in rich media and in defined minimal media. Bacterial growth at 37°C in (A) cysteine-enriched tryptic soy broth and (B) Chamberlain's minimal defined media is shown for wild-type F. novicida (circles), FTN_0096 (Δ96, squares), and FTN_1133 (Δ1133 triangles). Data shown is representative of at least three independent experiments. (TIF) [file pone.0024201.s001.tif]

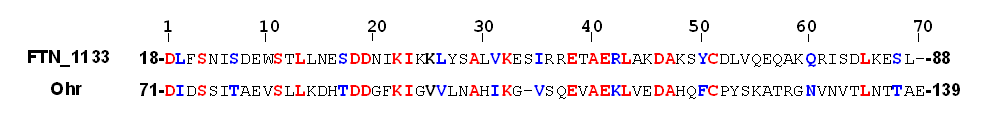

Supplement: Figure S2 — FTN_1133 has similarity to the organic hydroperoxide resistance protein Ohr. The sequences for F. novicida FTN_1133 (a.a. 18 – 86) and Bacillus megaterium Ohr (a.a. 71 – 139) were aligned using CLUSTALW (http://www.ebi.ac.uk/Tools/msa/clustalw2/). Identical residues are highlighted in red and similar residues are highlighted in blue. The sequences have 28.6% identity and 42.9% similarity. (TIF) [file pone.0024201.s002.tif]
