# Supplementary material for: Macrophage Replication Screen Identifies a Novel Francisella Hydroperoxide Resistance Protein Involved in Virulence
Source: PLoS One. 2011 Sep 6;6(9):e24201. doi: 10.1371/journal.pone.0024201 (PMC3167825; doi:10.1371/journal.pone.0024201)
Supplement: Table S1 — Full list of transposon mutant replication phenotypes in RAW264.7 macrophages. Strains highlighted in bold were attenuated for intracellular replication. Each transposon mutant was screened 2–3 times and each time the fold replication of the mutant was compared to the fold replication of wild-type (fold Mut/ fold WT). AVG Mut/WT is the average of these ratios from all experiments for each mutant. Strain names, plate number, and well location are as annotated in the two-allele transposon mutant library from Gallagher, et al. (DOC) [file pone.0024201.s003.doc]

| **FTN locus** | **FTT locus** | **Gene name** | **Gene Description** | **AVG Mut/WT** | **Strain Name** | **Plate Number** | **Well** | **Mammalian *in vivo* attenuation references** | | **Mammalian *in vitro* attenuation references** |
| --- | --- | --- | --- | --- | --- | --- | --- | --- | --- | --- |
| **FTN_0019** | **FTT1665** | ***pyrB*** | **aspartate carbamoyltransferase** | **0.03** | **tnfn1_pw060510p01q109** | **NR-8063** | **A02** |  | |  |
| **FTN_0019** | **FTT1665** | ***pyrB*** | **aspartate carbamoyltransferase** | **0.06** | **tnfn1_pw060323p03q119** | **NR-8037** | **C03** |  | |  |
| **FTN_0020** | **FTT1664** | ***carB*** | **carbamoyl-phosphate synthase large chain** | **0.18** | **tnfn1_pw060328p01q118** | **NR-8043** | **B03** |  | |  |
| **FTN_0020** | **FTT1664** | ***carB*** | **carbamoyl-phosphate synthase large chain** | **0.09** | **tnfn1_pw060323p08q120** | **NR-8042** | **D03** |  | |  |
| **FTN_0021** | **FTT1663** | ***carA*** | **carbamoyl-phosphate synthase small chain** | **0.19** | **tnfn1_pw060510p02q160** | **NR-8064** | **D08** |  | |  |
| FTN_0021 | FTT1663 | *carA* | carbamoyl-phosphate synthase small chain | 0.50 | tnfn1_pw060328p04q165 | NR-8046 | A09 |  | |  |
| **FTN_0035** | **FTT1648c** | ***pyrF*** | **orotidine-5'-phosphate decarboxylase** | **0.05** | **tnfn1_pw060510p01q129** | **NR-8063** | **E04** |  | |  |
| **FTN_0035** | **FTT1648c** | ***pyrF*** | **orotidine-5'-phosphate decarboxylase** | **0.09** | **tnfn1_pw060323p04q176** | **NR-8038** | **D10** |  | |  |
| **FTN_0036** | **FTT1647c** | ***pyrD*** | **dihydroorotate oxidase** | **0.22** | **tnfn1_pw060510p04q195** | **NR-8066** | **G12** |  | |  |
| **FTN_0036** | **FTT1647c** | ***pyrD*** | **dihydroorotate oxidase** | **0.26** | **tnfn1_pw060323p03q144** | **NR-8037** | **D06** |  | |  |
| **FTN_0096** | **FTT1689c** | **NA** | **hypothetical protein** | **0.00** | **tnfn1_pw060328p06q161** | **NR-8048** | **E08** |  | |  |
| FTN_0096 | FTT1689c | NA | hypothetical protein | 0.65 | tnfn1_pw060419p03q167 | NR-8057 | C09 |  | |  |
| FTN_0097 | FTT1688 | NA | hydroxy/aromatic amino acid permease (HAAAP) family protein | 0.51 | tnfn1_pw060328p06q162 | NR-8048 | F08 |  | |  |
| FTN_0097 | FTT1688 | NA | hydroxy/aromatic amino acid permease (HAAAP) family protein | 1.43 | tnfn1_pw060420p01q180 | NR-8059 | H10 |  | |  |
| FTN_0098 | FTT1687c | *gidB* | glucose-inhibited cell division protein | 0.65 | tnfn1_pw060328p04q143 | NR-8046 | C06 |  | |  |
| FTN_0098 | FTT1687c | *gidB* | glucose-inhibited cell division protein | 1.50 | tnfn1_pw060419p01q136 | NR-8055 | D05 |  | |  |
| **FTN_0109** | **FTT1676** | **NA** | **hypothetical protein** | **0.07** | **tnfn1_pw060328p06q147** | **NR-8048** | **G06** |  | |  |
| FTN_0109 | FTT1676 | NA | hypothetical protein | 1.28 | tnfn1_pw060418p04q193 | NR-8054 | E12 |  | |  |
| FTN_0113 | FTT1672 | *ribC* | riboflavin synthase alpha chain | 0.71 | tnfn1_pw060420p02q191 | NR-8060 | C12 |  | |  |
| FTN_0113 | FTT1672 | *ribC* | riboflavin synthase alpha chain | 2.13 | tnfn1_pw060328p01q121 | NR-8043 | E03 |  | |  |
| FTN_0119 | FTT1747 | NA | conserved outer membrane protein of unknown function | 0.56 | tnfn1_pw060323p08q158 | NR-8042 | B08 |  | |  |
| FTN_0119 | FTT1747 | NA | conserved outer membrane protein of unknown function | 1.50 | tnfn1_pw060419p03q120 | NR-8057 | D03 |  | |  |
| FTN_0120 | FTT1748 | NA | rhodanese-related sulfurtransferase | 1.00 | tnfn1_pw060328p08q125 | NR-8050 | A04 |  | |  |
| FTN_0120 | FTT1748 | NA | rhodanese-related sulfurtransferase | 2.92 | tnfn1_pw060418p03q181 | NR-8053 | A11 |  | |  |
| FTN_0122 | FTT1750 | *recA* | recombinase A protein | 0.50 | tnfn1_pw060323p01q152 | NR-8035 | D07 |  | |  |
| FTN_0122 | FTT1750 | *recA* | recombinase A protein | 2.17 | tnfn1_pw060510p04q169 | NR-8066 | E09 |  | |  |
| FTN_0132 | FTT0256c | NA | hypothetical protein | 0.50 | tnfn1_pw060323p02q111 | NR-8036 | C02 |  | |  |
| FTN_0132 | FTT0256c | NA | hypothetical protein | 0.64 | tnfn1_pw060510p01q123 | NR-8063 | G03 |  | |  |
| **FTN_0177** | **FTT0203c** | ***purH*** | **AICAR transformylase/IMP cyclohydrolase** | **0.07** | **tnfn1_pw060328p04q192** | **NR-8046** | **D12** |  | |  |
| **FTN_0177** | **FTT0203c** | ***purH*** | **AICAR transformylase/IMP cyclohydrolase** | **0.07** | **tnfn1_pw060323p06q116** | **NR-8040** | **H02** |  | |  |
| FTN_0177 | FTT0203c | *purH* | AICAR transformylase/IMP cyclohydrolase | 1.00 | tnfn1_pw060510p03q162 | NR-8065 | F08 |  | |  |
| **FTN_0178** | **FTT0204** | ***purA*** | **adenylosuccinate synthetase** | **0.01** | **tnfn1_pw060323p03q133** | **NR-8037** | **A05** |  | |  |
| **FTN_0178** | **FTT0204** | ***purA*** | **adenylosuccinate synthetase** | **0.04** | **tnfn1_pw060510p03q187** | **NR-8065** | **G11** |  | |  |
| FTN_0183 | FTT0209c | NA | manganese/Zinc/Iron chelate uptake transporter family protein | 1.22 | tnfn1_pw060419p01q165 | NR-8058 | C01 |  | |  |
| FTN_0183 | FTT0209c | NA | manganese/Zinc/Iron chelate uptake transporter family protein | 1.75 | tnfn1_pw060419p01q165 | NR-8055 | A09 |  | |  |
| FTN_0196 | FTT0282 | *cyoB* | cytochrome bo terminal oxidase subunit I | 0.75 | tnfn1_pw060323p05q118 | NR-8039 | B03 |  | |  |
| FTN_0196 | FTT0282 | *cyoB* | cytochrome bo terminal oxidase subunit I | 1.00 | tnfn1_pw060418p02q121 | NR-8052 | E03 |  | |  |
| FTN_0197 | FTT0283 | *cyoC* | cytochrome bo terminal oxidase subunit III | 0.42 | tnfn1_pw060419p02q183 | NR-8056 | C11 |  | |  |
| FTN_0197 | FTT0283 | *cyoC* | cytochrome bo terminal oxidase subunit III | 0.90 | tnfn1_pw060328p08q152 | NR-8050 | D07 |  | |  |
| FTN_0198 | FTT0284 | *cyoD* | cytochrome bo terminal oxidase subunit IV | 0.85 | tnfn1_pw060420p03q154 | NR-8061 | F07 |  | |  |
| FTN_0198 | FTT0284 | *cyoD* | cytochrome bo terminal oxidase subunit IV | 1.00 | tnfn1_pw060328p04q194 | NR-8046 | F12 |  | |  |
| FTN_0202 | FTT0288c | *pdxY* | pyridoxal kinase | 1.46 | tnfn1_pw060418p04q144 | NR-8054 | D06 |  | |  |
| FTN_0210 | FTT0295 | NA | hypothetical protein | 0.57 | tnfn1_pw060510p01q155 | NR-8063 | G07 |  | |  |
| FTN_0210 | FTT0295 | NA | hypothetical protein | 0.81 | tnfn1_pw060323p08q153 | NR-8042 | E07 |  | |  |
| FTN_0210 | FTT0295 | NA | hypothetical protein | 0.81 | tnfn1_pw060323p08q152 | NR-8042 | D07 |  | |  |
| FTN_0211 | FTT0296 | *pcp* | pyrrolidone carboxylylate peptidase | 0.50 | tnfn1_pw060323p04q102 | NR-8038 | B01 |  | |  |
| FTN_0211 | FTT0296 | *pcp* | pyrrolidone carboxylylate peptidase | 2.92 | tnfn1_pw060418p03q177 | NR-8053 | E10 |  | |  |
| FTN_0265 | FTT0351 | *rplQ* | 50S ribosomal protein L17 | 0.50 | tnfn1_pw060323p03q185 | NR-8037 | E11 |  | |  |
| FTN_0266 | FTT0356 | *htpG* | chaperone Hsp90, heat shock protein HtpG | 0.52 | tnfn1_pw060328p08q114 | NR-8050 | F02 |  | |  |
| FTN_0266 | FTT0356 | *htpG* | chaperone Hsp90, heat shock protein HtpG | 0.62 | tnfn1_pw060328p02q157 | NR-8044 | A08 |  | |  |
| FTN_0289 | FTT1640c | *proQ* | activator of osmoprotectant transporter ProP | 0.86 | tnfn1_pw060419p04q137 | NR-8058 | E05 |  | |  |
| FTN_0296 | FTT1633c | *lysP* | lysine:H+ symporter | 0.64 | tnfn1_pw060328p04q193 | NR-8046 | E12 |  | |  |
| FTN_0296 | FTT1633c | *lysP* | lysine:H+ symporter | 0.70 | tnfn1_pw060419p04q126 | NR-8058 | B04 |  | |  |
| FTN_0296 | FTT1633c | *lysP* | lysine:H+ symporter | 1.39 | tnfn1_pw060419p04q133 | NR-8058 | A05 |  | |  |
| FTN_0297 | FTT1632c | NA | hypothetical protein | 0.72 | tnfn1_pw060420p04q134 | NR-8062 | B05 |  | |  |
| FTN_0297 | FTT1632c | NA | hypothetical protein | 0.81 | tnfn1_pw060323p08q103 | NR-8042 | C01 |  | |  |
| FTN_0298 | FTT1631c | *gplX* | fructose 1,6-bisphosphatase II | 1.00 | tnfn1_pw060419p02q168 | NR-8056 | D09 |  | |  |
| FTN_0298 | FTT1631c | *gplX* | fructose 1,6-bisphosphatase II | 1.50 | tnfn1_pw060328p05q168 | NR-8047 | D09 |  | |  |
| FTN_0325 | FTT1611 | NA | membrane protein of unknown function | 0.40 | tnfn1_pw060328p04q119 | NR-8046 | C03 |  | |  |
| FTN_0325 | FTT1611 | NA | membrane protein of unknown function | 1.42 | tnfn1_pw060418p03q169 | NR-8053 | E09 |  | |  |
| FTN_0330 | FTT1606 | *minD* | septum formation inhibitor-activating ATPase | 0.58 | tnfn1_pw060328p01q167 | NR-8043 | C09 |  | |  |
| FTN_0330 | FTT1606 | *minD* | septum formation inhibitor-activating ATPase | 0.69 | tnfn1_pw060328p01q104 | NR-8043 | D01 |  | |  |
| FTN_0331 | FTT1605 | *minC* | septum formation inhibitor | 0.56 | tnfn1_pw060323p08q146 | NR-8042 | F06 |  | |  |
| FTN_0331 | FTT1605 | *minC* | septum formation inhibitor | 1.09 | tnfn1_pw060420p02q170 | NR-8060 | F09 |  | |  |
| FTN_0337 | FTT1600c | *fumA* | fumarate hydratase, class I | 0.58 | tnfn1_pw060328p03q144 | NR-8045 | D06 |  | |  |
| FTN_0337 | FTT1600c | *fumA* | fumarate hydratase, class I | 1.11 | tnfn1_pw060420p01q158 | NR-8059 | B08 |  | |  |
| FTN_0358 | FTT0843 | NA | tRNA-methylthiotransferase MiaB protein | 0.55 | tnfn1_pw060328p06q121 | NR-8048 | E03 |  | |  |
| FTN_0358 | FTT0843 | NA | tRNA-methylthiotransferase MiaB protein | 0.69 | tnfn1_pw060328p03q179 | NR-8045 | G10 |  | |  |
| FTN_0358 | FTT0843 | NA | tRNA-methylthiotransferase MiaB protein | 1.75 | tnfn1_pw060419p01q169 | NR-8055 | E09 |  | |  |
| FTN_0407 | FTT0881c | NA | amino acid transporter (AAT) family protein | 0.63 | tnfn1_pw060323p07q130 | NR-8041 | F04 |  | |  |
| FTN_0407 | FTT0881c | NA | amino acid transporter (AAT) family protein | 1.75 | tnfn1_pw060418p03q155 | NR-8053 | G07 |  | |  |
| FTN_0410 | FTT0884c | NA | aspartate/tyrosine/aromatic aminotransferase | 0.50 | tnfn1_pw060323p01q142 | NR-8035 | B06 |  | |  |
| FTN_0410 | FTT0884c | NA | aspartate/tyrosine/aromatic aminotransferase | 2.14 | tnfn1_pw060420p01q150 | NR-8059 | B07 |  | |  |
| FTN_0416 | FTT0891 | *lpxE* | lipid A 1-phosphatase | 0.56 | tnfn1_pw060328p01q185 | NR-8043 | E11 |  | |  |
| FTN_0416 | FTT0891 | *lpxE* | lipid A 1-phosphatase | 1.43 | tnfn1_pw060418p04q182 | NR-8054 | B11 |  | |  |
| FTN_0417 | FTT0892 | *folD* | methyleneTHF enzyme/ methenyltetrahydrofolate cyclohydrolase/ methylenetetrahydrofolate dehydrogenase | 0.48 | tnfn1_pw060328p05q131 | NR-8047 | G04 |  | |  |
| **FTN_0419** | **FTT0893** | ***purM*** | **phosphoribosylformylglycinamide cyclo-ligase** | **0.05** | **tnfn1_pw060323p08q193** | **NR-8042** | **E12** |  | |  |
| FTN_0419 | FTT0893 | *purM* | phosphoribosylformylglycinamide cyclo-ligase | 0.35 | tnfn1_pw060510p02q136 | NR-8064 | D05 |  | |  |
| FTN_0421 | FTT0895 | *purN* | phosphoribosylglycinamide formyltransferase | 1.08 | tnfn1_pw060510p02q128 | NR-8064 | D04 |  | |  |
| **FTN_0422** | **FTT0896** | ***purE*** | **N5-carboxyaminoimidazole ribonucleotide mutase** | **0.03** | **tnfn1_pw060323p08q194** | **NR-8042** | **F12** |  | |  |
| FTN_0422 | FTT0896 | *purE* | N5-carboxyaminoimidazole ribonucleotide mutase | 1.67 | tnfn1_pw060510p04q172 | NR-8066 | H09 |  | |  |
| FTN_0427 | FTT0901 | NA | lipoprotein of unknown function | 1.67 | tnfn1_pw060418p04q133 | NR-8054 | A05 |  | |  |
| FTN_0429 | FTT0903 | NA | hypothetical protein | 0.99 | tnfn1_pw060328p06q195 | NR-8048 | G12 |  | |  |
| FTN_0429 | FTT0903 | NA | hypothetical protein | 1.42 | tnfn1_pw060418p03q178 | NR-8053 | F10 |  | |  |
| **FTN_0430** | **FTT0904** | **NA** | **hypothetical protein** | **0.30** | **tnfn1_pw060323p06q169** | **NR-8040** | **E09** |  | |  |
| FTN_0430 | FTT0904 | NA | hypothetical protein | 1.00 | tnfn1_pw060419p03q130 | NR-8057 | F04 |  | |  |
| FTN_0431 | FTT0905 | NA | hypothetical membrane protein | 0.71 | tnfn1_pw060420p02q140 | NR-8060 | H05 |  | |  |
| FTN_0431 | FTT0905 | NA | hypothetical membrane protein | 2.13 | tnfn1_pw060328p02q173 | NR-8044 | A10 |  | |  |
| FTN_0434 | FTT0908 | *parB* | chromosome partition protein B | 1.83 | tnfn1_pw060510p03q196 | NR-8065 | H12 |  | |  |
| FTN_0436 | FTT0910 | NA | lipolytic enzyme | 0.38 | tnfn1_pw060420p04q119 | NR-8062 | C03 |  | |  |
| FTN_0436 | FTT0910 | NA | lipolytic enzyme | 0.65 | tnfn1_pw060323p06q132 | NR-8040 | H04 |  | |  |
| FTN_0444 | FTT0918 | NA | membrane protein of unknown function | 1.06 | tnfn1_pw060420p03q175 | NR-8061 | C10 |  | |  |
| FTN_0444 | FTT0918 | NA | membrane protein of unknown function | 1.50 | tnfn1_pw060328p05q119 | NR-8047 | C03 |  | |  |
| FTN_0487 | FTT0390c | NA | 30S ribosomal protein S21 | 0.50 | tnfn1_pw060323p04q101 | NR-8038 | A01 |  | |  |
| FTN_0494 | FTT0398c | NA | hypothetical membrane protein | 0.50 | tnfn1_pw060323p02q112 | NR-8036 | D02 |  | |  |
| FTN_0494 | FTT0398c | NA | hypothetical membrane protein | 0.61 | tnfn1_pw060510p01q167 | NR-8063 | C09 |  | |  |
| FTN_0494 | FTT0398c | NA | hypothetical membrane protein | 1.50 | tnfn1_pw060419p03q142 | NR-8057 | B06 |  | |  |
| FTN_0495 | FTT0399c | NA | BNR/Asp-box repeat protein | 0.44 | tnfn1_pw060328p06q124 | NR-8048 | H03 |  | |  |
| FTN_0495 | FTT0399c | NA | BNR/Asp-box repeat protein | 0.89 | tnfn1_pw060420p03q136 | NR-8061 | D05 |  | |  |
| FTN_0504 | FTT0406 | NA | lysine decarboxylase | 1.00 | tnfn1_pw060323p05q182 | NR-8039 | B11 |  | |  |
| FTN_0504 | FTT0406 | NA | lysine decarboxylase | 1.67 | tnfn1_pw060510p04q152 | NR-8066 | D07 |  | |  |
| FTN_0505 | FTT0407 | *gcvT* | glycine cleavage complex protein T | 0.50 | tnfn1_pw060323p03q128 | NR-8037 | D04 |  | |  |
| FTN_0505 | FTT0407 | *gcvT* | glycine cleavage complex protein T | 0.50 | tnfn1_pw060323p07q143 | NR-8041 | C06 |  | |  |
| FTN_0505 | FTT0407 | *gcvT* | glycine cleavage complex protein T | 0.70 | tnfn1_pw060328p03q121 | NR-8045 | E03 |  | |  |
| FTN_0506 | FTT0408 | *gcvH* | glycine cleavage system H protein | 1.17 | tnfn1_pw060420p03q135 | NR-8061 | C05 |  | |  |
| FTN_0507 | FTT0409 | *gcvP1* | glycine cleavage system P protein, subunit 1 | 0.53 | tnfn1_pw060510p01q124 | NR-8063 | H03 |  | |  |
| FTN_0507 | FTT0409 | *gcvP1* | glycine cleavage system P protein, subunit 1 | 0.58 | tnfn1_pw060323p06q129 | NR-8040 | E04 |  | |  |
| FTN_0513 | FTT0413c | *glgB* | 1,4-alpha-glucan branching enzyme | 0.50 | tnfn1_pw060510p04q104 | NR-8066 | D01 |  | |  |
| FTN_0513 | FTT0413c | *glgB* | 1,4-alpha-glucan branching enzyme | 1.60 | tnfn1_pw060323p05q194 | NR-8039 | F12 |  | |  |
| FTN_0514 | FTT0414 | *pgm* | phosphoglucomutase | 0.50 | tnfn1_pw060510p02q132 | NR-8064 | H04 |  | |  |
| FTN_0514 | FTT0414 | *pgm* | phosphoglucomutase | 1.17 | tnfn1_pw060328p03q191 | NR-8045 | C12 |  | |  |
| FTN_0515 | FTT0415 | *glgC* | glucose-1-phosphate adenylyltransferase | 1.00 | tnfn1_pw060510p03q111 | NR-8065 | C02 |  | |  |
| FTN_0515 | FTT0415 | *glgC* | glucose-1-phosphate adenylyltransferase | 1.17 | tnfn1_pw060328p04q138 | NR-8046 | F05 |  | |  |
| FTN_0534 | FTT0443 | NA | hypothetical protein | 1.33 | tnfn1_pw060328p05q130 | NR-8047 | F04 |  | |  |
| FTN_0534 | FTT0443 | NA | hypothetical protein | 1.50 | tnfn1_pw060418p04q140 | NR-8054 | H05 |  | |  |
| FTN_0535 | FTT0444 | NA | drug:H+ antiporter-1 (DHA1) family protein | 0.67 | tnfn1_pw060323p04q126 | NR-8038 | B04 |  | |  |
| FTN_0535 | FTT0444 | NA | drug:H+ antiporter-1 (DHA1) family protein | 1.44 | tnfn1_pw060418p03q165 | NR-8053 | A09 |  | |  |
| **FTN_0544** | **FTT0453c** | **NA** | **hypothetical protein** | **0.17** | **tnfn1_pw060418p02q107** | **NR-8052** | **G01** |  | |  |
| FTN_0544 | FTT0453c | NA | hypothetical protein | 0.65 | tnfn1_pw060419p01q114 | NR-8055 | F02 |  | |  |
| FTN_0544 | FTT0453c | NA | hypothetical protein | 0.94 | tnfn1_pw060420p04q160 | NR-8062 | D08 |  | |  |
| FTN_0545 | FTT0454 | NA | glycosyl transferase, group 2 | 0.34 | tnfn1_pw060323p06q168 | NR-8040 | D09 |  | |  |
| FTN_0545 | FTT0454 | NA | glycosyl transferase, group 2 | 1.00 | tnfn1_pw060419p01q187 | NR-8055 | G11 |  | |  |
| **FTN_0546** | **FTT0455c** | ***flmK*** | **dolichyl-phosphate-mannose-protein mannosyltransferase family protein** | **0.14** | **tnfn1_pw060420p02q141** | **NR-8060** | **A06** |  | |  |
| **FTN_0546** | **FTT0455c** | ***flmK*** | **dolichyl-phosphate-mannose-protein mannosyltransferase family protein** | **0.15** | **tnfn1_pw060328p08q168** | **NR-8050** | **D09** |  | |  |
| FTN_0554 | FTT0463 | NA | RNA methyltransferase, SpoU family | 1.67 | tnfn1_pw060510p04q133 | NR-8066 | A05 |  | |  |
| FTN_0560 | FTT0469 | *ksgA* | dimethyladenosine transferase | 1.00 | tnfn1_pw060419p02q135 | NR-8056 | C05 |  | |  |
| FTN_0560 | FTT0469 | *ksgA* | dimethyladenosine transferase | 1.67 | tnfn1_pw060418p03q144 | NR-8053 | D06 |  | |  |
| FTN_0561 | FTT0470 | *apaH* | diadenosine tetraphosphatase | 1.50 | tnfn1_pw060419p02q156 | NR-8056 | H07 |  | |  |
| **FTN_0593** | **FTT0503c** | ***sucD*** | **succinyl-CoA synthetase, alpha subunit** | **0.06** | **tnfn1_pw060328p02q141** | **NR-8044** | **A06** |  | |  |
| FTN_0593 | FTT0503c | *sucD* | succinyl-CoA synthetase, alpha subunit | 1.00 | tnfn1_pw060419p03q116 | NR-8057 | H02 |  | |  |
| FTN_0593 | FTT0503c | *sucD* | succinyl-CoA synthetase, alpha subunit | 1.19 | tnfn1_pw060420p03q166 | NR-8061 | B09 |  | |  |
| **FTN_0594** | **FTT0504c** | ***sucC*** | **succinyl-CoA synthetase, beta chain** | **0.10** | **tnfn1_pw060323p06q147** | **NR-8040** | **G06** |  | |  |
| FTN_0594 | FTT0504c | *sucC* | succinyl-CoA synthetase, beta chain | 1.50 | tnfn1_pw060419p03q113 | NR-8057 | E02 |  | |  |
| **FTN_0599** | **FTT0509c** | **NA** | **hypothetical protein** | **0.25** | **tnfn1_pw060328p06q173** | **NR-8048** | **A10** |  | |  |
| FTN_0599 | FTT0509c | NA | hypothetical protein | 0.48 | tnfn1_pw060418p02q187 | NR-8052 | G11 |  | |  |
| FTN_0599 | FTT0509c | NA | hypothetical protein | 0.83 | tnfn1_pw060510p04q147 | NR-8066 | G06 |  | |  |
| FTN_0620 | FTT0708 | NA | major facilitator superfamily (MFS) transport protein | 0.50 | tnfn1_pw060323p01q145 | NR-8035 | E06 |  | |  |
| FTN_0620 | FTT0708 | NA | major facilitator superfamily (MFS) transport protein | 1.18 | tnfn1_pw060419p04q167 | NR-8058 | C09 |  | |  |
| FTN_0624 | FTT0712c | NA | serine permease | 0.63 | tnfn1_pw060510p02q156 | NR-8064 | H07 |  | |  |
| FTN_0624 | FTT0712c | NA | serine permease | 1.00 | tnfn1_pw060323p06q164 | NR-8040 | H08 |  | |  |
| FTN_0624 | FTT0712c | NA | serine permease | 2.50 | tnfn1_pw060418p04q157 | NR-8054 | A08 |  | |  |
| FTN_0633 | FTT0721c | *katG* | peroxidase/catalase | 0.50 | tnfn1_pw060418p02q128 | NR-8052 | D04 |  | |  |
| FTN_0633 | FTT0721c | *katG* | peroxidase/catalase | 0.90 | tnfn1_pw060323p06q133 | NR-8040 | A05 |  | |  |
| FTN_0643 | FTT1334c | NA | hypothetical protein | 0.81 | tnfn1_pw060418p01q154 | NR-8051 | F07 |  | |  |
| FTN_0643 | FTT1334c | NA | hypothetical protein | 1.00 | tnfn1_pw060323p08q109 | NR-8042 | A02 |  | |  |
| FTN_0643 | FTT1334c | NA | hypothetical protein | 1.75 | tnfn1_pw060419p01q157 | NR-8055 | A08 |  | |  |
| FTN_0651 | FTT1327 | *cdd* | cytidine deaminase | 1.00 | tnfn1_pw060418p01q120 | NR-8051 | D03 |  | |  |
| FTN_0651 | FTT1327 | *cdd* | cytidine deaminase | 1.75 | tnfn1_pw060419p01q168 | NR-8055 | D09 |  | |  |
| FTN_0664 | FTT1314 | *fimT* | Type IV pili, pilus assembly protein | 0.38 | tnfn1_pw060328p03q183 | NR-8045 | C11 |  | |  |
| FTN_0664 | FTT1314 | *fimT* | Type IV pili, pilus assembly protein | 1.50 | tnfn1_pw060419p03q141 | NR-8057 | A06 |  | |  |
| FTN_0666 | FTT1312 | *uvrA* | excinuclease ABC, subunit A | 0.70 | tnfn1_pw060328p03q169 | NR-8045 | E09 |  | |  |
| FTN_0666 | FTT1312 | *uvrA* | excinuclease ABC, subunit A | 1.67 | tnfn1_pw060510p04q168 | NR-8066 | D09 |  | |  |
| FTN_0669 | FTT0766 | *deoD* | purine nucleoside phosphorylase | 0.50 | tnfn1_pw060510p03q113 | NR-8065 | E02 |  | |  |
| FTN_0669 | FTT0766 | *deoD* | purine nucleoside phosphorylase | 1.17 | tnfn1_pw060323p04q172 | NR-8038 | H09 |  | |  |
| FTN_0672 | FTT0769 | *secA* | preprotein translocase, subunit A (ATPase, RNA helicase) | 0.53 | tnfn1_pw060328p04q123 | NR-8046 | G03 |  | |  |
| FTN_0672 | FTT0769 | *secA* | preprotein translocase, subunit A (ATPase, RNA helicase) | 0.62 | tnfn1_pw060418p02q130 | NR-8052 | F04 |  | |  |
| FTN_0689 | FTT1472 | *ppiC* | parvulin-like peptidyl-prolyl isomerase domain | 0.50 | tnfn1_pw060323p01q118 | NR-8035 | B03 |  | |  |
| FTN_0689 | FTT1472 | *ppiC* | parvulin-like peptidyl-prolyl isomerase domain | 0.88 | tnfn1_pw060420p01q160 | NR-8059 | D08 |  | |  |
| FTN_0690 | FTT1471 | *deaD* | DEAD-box subfamily ATP-dependent helicase | 0.81 | tnfn1_pw060323p07q195 | NR-8041 | G12 |  | |  |
| FTN_0690 | FTT1471 | *deaD* | DEAD-box subfamily ATP-dependent helicase | 2.38 | tnfn1_pw060420p02q160 | NR-8060 | D08 |  | |  |
| FTN_0719 | intergenic; FTT0747c/FTT0748 | NA | hypothetical protein | 0.50 | tnfn1_pw060323p05q171 | NR-8039 | G09 |  | |  |
| FTN_0719 | intergenic; FTT0747c/FTT0748 | NA | hypothetical protein | 0.67 | tnfn1_pw060328p05q133 | NR-8047 | A05 |  | |  |
| FTN_0720 | FTT0748 | NA | transcriptional regulator, IclR family | 1.22 | tnfn1_pw060418p04q107 | NR-8054 | G01 |  | |  |
| FTN_0728 | FTT0756 | NA | predicted Co/Zn/Cd cation transporter | 1.04 | tnfn1_pw060328p06q132 | NR-8048 | H04 |  | |  |
| FTN_0728 | FTT0748 | NA | predicted Co/Zn/Cd cation transporter | 1.17 | tnfn1_pw060328p05q123 | NR-8047 | G03 |  | |  |
| FTN_0728 | FTT0756 | NA | predicted Co/Zn/Cd cation transporter | 1.44 | tnfn1_pw060418p03q149 | NR-8053 | A07 |  | |  |
| FTN_0731 | FTT0759 | NA | hypothetical protein | 0.50 | tnfn1_pw060323p03q186 | NR-8037 | F11 |  | |  |
| FTN_0731 | FTT0759 | NA | hypothetical protein | 0.63 | tnfn1_pw060328p02q104 | NR-8044 | D01 |  | |  |
| FTN_0756 | FTT0583 | *fopA* | OmpA family protein | 0.39 | tnfn1_pw060323p04q187 | NR-8038 | G11 |  | |  |
| FTN_0756 | FTT0583 | *fopA* | OmpA family protein | 0.49 | tnfn1_pw060510p01q195 | NR-8063 | G12 |  | |  |
| FTN_0756 | FTT0583 | *fopA* | OmpA family protein | 0.50 | tnfn1_pw060420p03q180 | NR-8061 | H10 |  | |  |
| FTN_0757 | FTT0584 | NA | membrane protein of unknown function | 0.75 | tnfn1_pw060419p02q184 | NR-8056 | D11 |  | |  |
| FTN_0757 | FTT0584 | NA | membrane protein of unknown function | 0.82 | tnfn1_pw060419p04q172 | NR-8058 | H09 |  | |  |
| FTN_0771 | FTT1103 | NA | protein-disulfide isomerase | 0.45 | tnfn1_pw060419p04q194 | NR-8058 | F12 |  | |  |
| FTN_0772 | FTT1102 | NA | hypothetical protein | 0.74 | tnfn1_pw060419p02q111 | NR-8056 | C02 |  | |  |
| FTN_0806 | FTT0928c | NA | glycosyl hydrolase family 3 | 0.50 | tnfn1_pw060323p02q108 | NR-8036 | H01 |  | |  |
| FTN_0806 | FTT0928c | NA | glycosyl hydrolase family 3 | 1.00 | tnfn1_pw060510p03q119 | NR-8065 | C03 |  | |  |
| **FTN_0812** | **FTT0934c** | ***bioD*** | **dethiobiotin synthetase** | **0.05** | **tnfn1_pw060323p01q189** | **NR-8035** | **A12** |  | |  |
| FTN_0812 | FTT0934c | *bioD* | dethiobiotin synthetase | 0.44 | tnfn1_pw060328p08q164 | NR-8050 | H08 |  | |  |
| FTN_0812 | FTT0934c | *bioD* | dethiobiotin synthetase | 0.56 | tnfn1_pw060418p01q175 | NR-8051 | C10 |  | |  |
| **FTN_0813** | **FTT0935c** | ***bioC*** | **biotin synthesis protein BioC** | **0.05** | **tnfn1_pw060323p04q196** | **NR-8038** | **H12** |  | |  |
| **FTN_0814** | **FTT0936c** | ***bioF*** | **8-amino-7-oxononanoate synthase** | **0.28** | **tnfn1_pw060419p02q138** | **NR-8056** | **F05** |  | |  |
| FTN_0814 | FTT0936c | *bioF* | 8-amino-7-oxononanoate synthase | 0.55 | tnfn1_pw060323p07q185 | NR-8041 | E11 |  | |  |
| **FTN_0815** | **FTT0937c** | ***bioB*** | **biotin synthase** | **0.05** | **tnfn1_pw060323p03q139** | **NR-8037** | **G05** |  | |  |
| FTN_0815 | FTT0937c | *bioB* | biotin synthase | 0.55 | tnfn1_pw060419p02q174 | NR-8056 | B10 |  | |  |
| **FTN_0816** | **FTT0938** | ***bioA*** | **adenosylmethionine-8-amino-7-oxononanoate aminotransferase** | **0.12** | **tnfn1_pw060328p05q108** | **NR-8047** | **H01** |  | |  |
| FTN_0816 | FTT0938 | *bioA* | adenosylmethionine-8-amino-7-oxononanoate aminotransferase | 0.58 | tnfn1_pw060420p02q145 | NR-8060 | E06 |  | |  |
| FTN_0817 | FTT0940c | NA | hypothetical protein | 0.88 | tnfn1_pw060323p06q170 | NR-8040 | F09 |  | |  |
| FTN_0817 | FTT0940c | NA | hypothetical protein | 1.00 | tnfn1_pw060418p01q141 | NR-8051 | A06 |  | |  |
| **FTN_0818** | **FTT0941c** | **NA** | **lipase/esterase** | **0.02** | **tnfn1_pw060323p02q160** | **NR-8036** | **D08** |  | |  |
| **FTN_0818** | **FTT0941c** | **NA** | **lipase/esterase** | **0.03** | **tnfn1_pw060323p02q159** | **NR-8036** | **C08** |  | |  |
| **FTN_0818** | **FTT0941c** | **NA** | **lipase/esterase** | **0.27** | **tnfn1_pw060328p04q175** | **NR-8046** | **C10** |  | |  |
| FTN_0818 | FTT0941c | NA | lipase/esterase | 0.36 | tnfn1_pw060328p04q172 | NR-8046 | H09 |  | |  |
| FTN_0821 | FTT0944 | NA | AMP-binding enzyme | 0.39 | tnfn1_pw060420p04q157 | NR-8062 | A08 |  | |  |
| FTN_0821 | FTT0944 | NA | AMP-binding enzyme | 5.00 | tnfn1_pw060323p02q137 | NR-8036 | E05 |  | |  |
| FTN_0822 | FTT0945 | NA | para-aminobenzoate synthase component I | 0.57 | tnfn1_pw060323p06q143 | NR-8040 | C06 |  | |  |
| FTN_0822 | FTT0945 | NA | para-aminobenzoate synthase component I | 0.69 | tnfn1_pw060420p04q108 | NR-8062 | H01 |  | |  |
| FTN_0823 | FTT0946 | *pabA* | para-aminobenzoate synthase component II | 0.50 | tnfn1_pw060323p01q148 | NR-8035 | H06 |  | |  |
| FTN_0823 | FTT0946 | *pabA* | para-aminobenzoate synthase component II | 1.50 | tnfn1_pw060418p02q134 | NR-8052 | B05 |  | |  |
| FTN_0842 | FTT0963c | *aroG* | phospho-2-dehydro-3-deoxyheptonate aldolase | 0.81 | tnfn1_pw060510p04q131 | NR-8066 | G04 |  | |  |
| FTN_0842 | FTT0963c | *aroG* | phospho-2-dehydro-3-deoxyheptonate aldolase | 1.50 | tnfn1_pw060323p07q179 | NR-8041 | G10 |  | |  |
| **FTN_0848** | **FTT0968c** | **NA** | **amino acid antiporter** | **0.02** | **tnfn1_pw060323p02q136** | **NR-8036** | **D05** |  | |  |
| FTN_0848 | FTT0968c | NA | amino acid antiporter | 0.55 | tnfn1_pw060418p04q151 | NR-8054 | C07 |  | |  |
| FTN_0855 | FTT0975 | NA | hypothetical protein | 1.00 | tnfn1_pw060420p04q176 | NR-8062 | D10 |  | |  |
| FTN_0855 | FTT0975 | NA | hypothetical protein | 1.42 | tnfn1_pw060328p05q125 | NR-8047 | A04 |  | |  |
| FTN_0893 | FTT1015 | NA | hypothetical protein | 0.50 | tnfn1_pw060323p03q193 | NR-8037 | E12 |  | |  |
| FTN_0893 | FTT1015 | NA | hypothetical protein | 1.17 | tnfn1_pw060420p03q160 | NR-8061 | D08 |  | |  |
| FTN_0925 | FTT1047c | NA | hypothetical protein | 0.54 | tnfn1_pw060328p03q147 | NR-8045 | G06 |  | |  |
| FTN_0925 | FTT1048c | NA | hypothetical protein | 2.75 | tnfn1_pw060419p04q188 | NR-8058 | H11 |  | |  |
| FTN_0945 | FTT1056c | *rsuA* | 16S rRNA pseudouridine synthase | 0.78 | tnfn1_pw060510p02q145 | NR-8064 | E06 |  | |  |
| FTN_0945 | FTT1056c | *rsuA* | 16S rRNA pseudouridine synthase | 1.17 | tnfn1_pw060328p05q161 | NR-8047 | E08 |  | |  |
| FTN_0998 | FTT0685c | NA | potassium channel protein | 0.67 | tnfn1_pw060420p01q191 | NR-8059 | C12 |  | |  |
| FTN_0998 | FTT0685c | NA | potassium channel protein | 0.84 | tnfn1_pw060323p05q184 | NR-8039 | D11 |  | |  |
| FTN_0999 | FTT0684c | *udhA* | soluble pyridine nucleotide transhydrogenase | 0.50 | tnfn1_pw060323p01q131 | NR-8035 | G04 |  | |  |
| FTN_0999 | FTT0684c | *udhA* | soluble pyridine nucleotide transhydrogenase | 1.31 | tnfn1_pw060418p03q163 | NR-8053 | G08 |  | |  |
| FTN_1007 | FTT0675 | *rplY* | 50S ribosomal protein L25 | 0.50 | tnfn1_pw060323p02q164 | NR-8036 | H08 |  | |  |
| FTN_1016 | FTT0667 | NA | hypothetical protein | 0.72 | tnfn1_pw060328p01q165 | NR-8043 | A09 |  | |  |
| FTN_1016 | FTT0667 | NA | hypothetical protein | 1.50 | tnfn1_pw060419p02q170 | NR-8056 | F09 |  | |  |
| FTN_1029 | FTT0654 | *elbB* | DJ-1/PfpI family protein | 0.50 | tnfn1_pw060323p04q186 | NR-8038 | F11 |  | |  |
| FTN_1029 | FTT0654 | *elbB* | DJ-1/PfpI family protein | 1.50 | tnfn1_pw060419p03q146 | NR-8057 | F06 |  | |  |
| FTN_1038 | FTT0645c | NA | hypothetical protein | 0.70 | tnfn1_pw060419p02q108 | NR-8056 | H01 |  | |  |
| FTN_1048 | FTT0633 | *hflK* | HflK-HflC membrane protein complex, HflK | 0.33 | tnfn1_pw060420p01q192 | NR-8059 | D12 |  | |  |
| FTN_1048 | FTT0633 | *hflK* | HflK-HflC membrane protein complex, HflK | 0.68 | tnfn1_pw060323p07q121 | NR-8041 | E03 |  | |  |
| FTN_1050 | FTT0631 | *hflX* | protease, GTP-binding subunit | 0.47 | tnfn1_pw060419p04q125 | NR-8058 | A04 |  | |  |
| FTN_1050 | FTT0631 | *hflX* | protease, GTP-binding subunit | 0.67 | tnfn1_pw060323p08q161 | NR-8042 | E08 |  | |  |
| FTN_1055 | FTT0626 | *lon* | DNA-binding, ATP-dependent protease La | 0.67 | tnfn1_pw060418p01q153 | NR-8051 | E07 |  | |  |
| FTN_1056 | FTT0625 | *clpX* | ATP-dependent Clp protease subunit X | 0.65 | tnfn1_pw060420p03q172 | NR-8061 | H09 |  | |  |
| FTN_1057 | FTT0624 | *clpP* | ATP-dependent Clp protease subunit P | 0.55 | tnfn1_pw060328p02q113 | NR-8044 | E02 |  | |  |
| FTN_1057 | FTT0624 | *clpP* | ATP-dependent Clp protease subunit P | 1.00 | tnfn1_pw060419p03q136 | NR-8057 | D05 |  | |  |
| FTN_1058 | FTT0623 | *tig* | trigger factor (TF) protein (peptidyl-prolyl cis/trans isomerase) | 0.40 | tnfn1_pw060323p04q107 | NR-8038 | G01 |  | |  |
| FTN_1058 | FTT0623 | *tig* | trigger factor (TF) protein (peptidyl-prolyl cis/trans isomerase) | 1.00 | tnfn1_pw060418p03q174 | NR-8053 | B10 |  | |  |
| FTN_1058 | FTT0623 | *tig* | trigger factor (TF) protein (peptidyl-prolyl cis/trans isomerase) | 1.21 | tnfn1_pw060323p06q141 | NR-8040 | A06 |  | |  |
| FTN_1064 | FTT0617c | NA | PhoH family protein, putative ATPase | 1.17 | tnfn1_pw060420p03q121 | NR-8061 | E03 |  | |  |
| FTN_1066 | FTT0615c | NA | transporter-associated protein, HlyC/CorC family | 0.44 | tnfn1_pw060328p03q134 | NR-8045 | B05 |  | |  |
| FTN_1066 | FTT0615c | NA | transporter-associated protein, HlyC/CorC family | 0.89 | tnfn1_pw060420p04q123 | NR-8062 | G03 |  | |  |
| FTN_1090 | FTT0589 | NA | membrane protein of unknown function | 0.50 | tnfn1_pw060323p02q135 | NR-8036 | C05 |  | |  |
| FTN_1090 | FTT0589 | NA | membrane protein of unknown function | 1.00 | tnfn1_pw060418p02q191 | NR-8052 | C12 |  | |  |
| FTN_1091 | FTT0588 | *aroA* | 3-phosphoshikimate 1-carboxyvinyltransferase | 0.42 | tnfn1_pw060328p04q101 | NR-8046 | A01 |  | |  |
| FTN_1091 | FTT0588 | *aroA* | 3-phosphoshikimate 1-carboxyvinyltransferase | 0.72 | tnfn1_pw060510p02q161 | NR-8064 | E08 |  | |  |
| FTN_1091 | FTT0588 | *aroA* | 3-phosphoshikimate 1-carboxyvinyltransferase | 0.77 | tnfn1_pw060419p04q168 | NR-8058 | D09 |  | |  |
| FTN_1097 | FTT1117c | NA | isochorismatase family protein | 1.17 | tnfn1_pw060418p02q101 | NR-8052 | A01 |  | |  |
| FTN_1097 | FTT1117c | NA | isochorismatase family protein | 2.00 | tnfn1_pw060418p04q122 | NR-8054 | F03 |  | |  |
| FTN_1107 | FTT1125 | *metlQ* | methionine uptake transporter (MUT) family protein, membrane and periplasmic protein | 0.71 | tnfn1_pw060328p02q109 | NR-8044 | A02 |  | |  |
| FTN_1107 | FTT1125 | *metlQ* | methionine uptake transporter (MUT) family protein, membrane and periplasmic protein | 1.08 | tnfn1_pw060418p04q163 | NR-8054 | G08 |  | |  |
| FTN_1107 | FTT1125 | *metlQ* | methionine uptake transporter (MUT) family protein, membrane and periplasmic protein | 1.67 | tnfn1_pw060510p03q130 | NR-8065 | F04 |  | |  |
| FTN_1111 | FTT1129c | NA | Mur ligase family protein | 0.41 | tnfn1_pw060323p06q130 | NR-8040 | F04 |  | |  |
| FTN_1111 | FTT1129c | NA | Mur ligase family protein | 0.63 | tnfn1_pw060418p04q102 | NR-8054 | B01 |  | |  |
| FTN_1112 | FTT1130c | *cphA* | cyanophycin synthetase | 0.43 | tnfn1_pw060420p03q118 | NR-8061 | B03 |  | |  |
| FTN_1112 | FTT1130c | *cphA* | cyanophycin synthetase | 1.04 | tnfn1_pw060328p03q132 | NR-8045 | H04 |  | |  |
| FTN_1131 | FTT1150c | *putA* | bifunctional proline dehydrogenase, pyrroline-5-carboxylate dehydrogenase | 0.50 | tnfn1_pw060323p04q179 | NR-8038 | G10 |  | |  |
| FTN_1131 | FTT1150c | *putA* | bifunctional proline dehydrogenase, pyrroline-5-carboxylate dehydrogenase | 0.63 | tnfn1_pw060328p02q175 | NR-8044 | C10 |  | |  |
| **FTN_1133** | **FTT1152** | **NA** | **hypothetical protein** | **0.30** | **tnfn1_pw060328p04q129** | **NR-8046** | **E04** |  | |  |
| FTN_1133 | FTT1152 | NA | hypothetical protein | 0.37 | tnfn1_pw060420p01q163 | NR-8059 | G08 |  | |  |
| FTN_1146 | FTT1165c | NA | aspartate aminotransferase | 0.50 | tnfn1_pw060323p02q150 | NR-8036 | B07 |  | |  |
| FTN_1146 | FTT1165c | NA | aspartate aminotransferase | 1.50 | tnfn1_pw060419p03q174 | NR-8057 | B10 |  | |  |
| FTN_1157 | FTT1179 | NA | GTP binding translational elongation factor Tu and G family protein | 0.67 | tnfn1_pw060420p03q176 | NR-8061 | D10 |  | |  |
| FTN_1157 | FTT1179 | NA | GTP binding translational elongation factor Tu and G family protein | 0.89 | tnfn1_pw060323p07q186 | NR-8041 | F11 |  | |  |
| FTN_1159 | FTT1181 | *ggt* | gamma-glutamyltranspeptidase | 1.00 | tnfn1_pw060328p08q180 | NR-8050 | H10 |  | |  |
| FTN_1159 | FTT1181 | *ggt* | gamma-glutamyltranspeptidase | 1.00 | tnfn1_pw060418p01q146 | NR-8051 | F06 |  | |  |
| FTN_1199 | FTT0807 | NA | hypothetical protein | 0.67 | tnfn1_pw060323p04q135 | NR-8038 | C05 |  | |  |
| FTN_1199 | FTT0807 | NA | hypothetical protein | 1.22 | tnfn1_pw060418p04q179 | NR-8054 | G10 |  | |  |
| FTN_1200 | FTT0806 | *capC* | capsule biosynthesis protein CapC | 0.59 | tnfn1_pw060418p02q139 | NR-8052 | G05 |  | |  |
| FTN_1201 | FTT0805 | *capB* | capsule biosynthesis protein CapB | 0.63 | tnfn1_pw060328p02q103 | NR-8044 | C01 |  | |  |
| FTN_1201 | FTT0805 | *capB* | capsule biosynthesis protein CapB | 2.92 | tnfn1_pw060418p03q121 | NR-8053 | E03 |  | |  |
| FTN_1209 | FTT0802 | *cphB* | cyanophycinase | 0.50 | tnfn1_pw060323p04q177 | NR-8038 | E10 |  | |  |
| FTN_1209 | FTT0802 | *cphB* | cyanophycinase | 1.75 | tnfn1_pw060419p01q138 | NR-8055 | F05 |  | |  |
| FTN_1211 | FTT0800 | NA | haloacid dehalogenase-like hydrolase | 1.67 | tnfn1_pw060323p01q185 | NR-8035 | E11 |  | |  |
| FTN_1211 | FTT0800 | NA | haloacid dehalogenase-like hydrolase | 2.50 | tnfn1_pw060418p03q131 | NR-8053 | G04 |  | |  |
| FTN_1212 | FTT0799 | NA | glycosyl transferase, group 1 | 0.67 | tnfn1_pw060420p04q101 | NR-8062 | A01 |  | |  |
| FTN_1212 | FTT0799 | NA | glycosyl transferase, group 1 | 0.78 | tnfn1_pw060323p05q162 | NR-8039 | F08 |  | |  |
| FTN_1213 | FTT0798 | NA | glycosyl transferase, family 2 | 0.50 | tnfn1_pw060323p03q152 | NR-8037 | D07 |  | |  |
| FTN_1213 | FTT0798 | NA | glycosyl transferase, family 2 | 1.17 | tnfn1_pw060510p04q120 | NR-8066 | D03 |  | |  |
| FTN_1214 | FTT0797 | NA | glycosyl transferase, family 2 | 0.83 | tnfn1_pw060328p06q149 | NR-8048 | A07 |  | |  |
| FTN_1214 | FTT0797 | NA | glycosyl transferase, family 2 | 0.90 | tnfn1_pw060418p03q150 | NR-8053 | B07 |  | |  |
| FTN_1214 | FTT0797 | NA | glycosyl transferase, family 2 | 0.92 | tnfn1_pw060510p01q174 | NR-8063 | B10 |  | |  |
| FTN_1217 | FTT0793 | NA | ATP-binding cassette (ABC) superfamily protein | 2.50 | tnfn1_pw060418p03q107 | NR-8053 | G01 |  | |  |
| FTN_1218 | FTT0792 | NA | glycosyl transferase, group 1 | 0.94 | tnfn1_pw060420p04q161 | NR-8062 | E08 |  | |  |
| FTN_1218 | FTT0792 | NA | glycosyl transferase, group 1 | 1.00 | tnfn1_pw060323p07q127 | NR-8041 | C04 |  | |  |
| FTN_1219 | FTT0791 | *galE* | UDP-glucose 4-epimerase | 1.08 | tnfn1_pw060328p08q135 | NR-8050 | C05 |  | |  |
| FTN_1219 | FTT0791 | *galE* | UDP-glucose 4-epimerase | 1.25 | tnfn1_pw060510p02q175 | NR-8064 | C10 |  | |  |
| FTN_1219 | FTT0791 | *galE* | UDP-glucose 4-epimerase | 1.80 | tnfn1_pw060510p02q177 | NR-8064 | E10 |  | |  |
| FTN_1220 | FTT0790 | NA | sugar transferase involved in lipopolysaccharide synthesis | 0.92 | tnfn1_pw060420p03q139 | NR-8061 | G05 |  | |  |
| FTN_1220 | FTT0790 | NA | sugar transferase involved in lipopolysaccharide synthesis | 1.17 | tnfn1_pw060323p06q191 | NR-8040 | C12 |  | |  |
| **FTN_1240** | **FTT1221** | **NA** | **BolA family protein** | **0.24** | **tnfn1_pw060419p04q183** | **NR-8049** | **E10** |  | |  |
| FTN_1242 | FTT1223 | NA | DedA family protein | 0.71 | tnfn1_pw060418p02q190 | NR-8052 | B12 |  | |  |
| FTN_1242 | FTT1223 | NA | DedA family protein | 0.90 | tnfn1_pw060328p01q163 | NR-8043 | G08 |  | |  |
| FTN_1252 | FTT1234 | NA | choloylglycine hydrolase family protein | 0.50 | tnfn1_pw060419p04q171 | NR-8058 | G09 |  | |  |
| FTN_1252 | FTT1234 | NA | choloylglycine hydrolase family protein | 0.78 | tnfn1_pw060323p04q169 | NR-8038 | E09 |  | |  |
| **FTN_1254** | **FTT1236** | **NA** | **hypothetical protein** | **0.20** | **tnfn1_pw060323p02q156** | **NR-8036** | **H07** |  | |  |
| **FTN_1254** | **FTT1236** | **NA** | **hypothetical protein** | **0.30** | **tnfn1_pw060323p02q155** | **NR-8036** | **G07** |  | |  |
| FTN_1254 | FTT1236 | NA | hypothetical protein | 0.47 | tnfn1_pw060510p04q135 | NR-8066 | C05 |  | |  |
| FTN_1254 | FTT1236 | NA | hypothetical protein | 1.00 | tnfn1_pw060510p03q135 | NR-8065 | C05 |  | |  |
| FTN_1255 | FTT1237 | NA | glycosyl transferase, family 8 | 1.00 | tnfn1_pw060323p05q183 | NR-8039 | C11 |  | |  |
| FTN_1256 | FTT1238c | NA | membrane protein of unknown function | 0.75 | tnfn1_pw060420p04q196 | NR-8062 | H12 |  | |  |
| FTN_1257 | FTT1239 | NA | membrane protein of unknown function | 0.50 | tnfn1_pw060323p03q102 | NR-8037 | B01 |  | |  |
| FTN_1257 | FTT1239 | NA | membrane protein of unknown function | 1.50 | tnfn1_pw060419p03q150 | NR-8057 | B07 |  | |  |
| FTN_1263 | FTT1244c | *comL* | competence lipoprotein | 0.76 | tnfn1_pw060328p06q171 | NR-8048 | G09 |  | |  |
| FTN_1263 | FTT1244c | *comL* | competence lipoprotein | 1.66 | tnfn1_pw060420p02q179 | NR-8060 | G10 |  | |  |
| FTN_1273 | FTT1254 | NA | long chain fatty acid CoA ligase | 0.52 | tnfn1_pw060510p04q149 | NR-8066 | A07 |  | |  |
| FTN_1273 | FTT1254 | NA | long chain fatty acid CoA ligase | 0.75 | tnfn1_pw060328p06q166 | NR-8048 | B09 |  | |  |
| FTN_1276 | FTT1257 | NA | membrane fusion protein | 1.19 | tnfn1_pw060420p03q109 | NR-8061 | A02 |  | |  |
| FTN_1276 | FTT1257 | NA | membrane fusion protein | 1.42 | tnfn1_pw060510p02q119 | NR-8064 | C03 |  | |  |
| FTN_1277 | FTT1258 | NA | outer membrane efflux protein | 0.36 | tnfn1_pw060510p01q136 | NR-8063 | D05 |  | |  |
| FTN_1277 | FTT1258 | NA | outer membrane efflux protein | 1.22 | tnfn1_pw060418p04q138 | NR-8054 | F05 |  | |  |
| FTN_1309 | FTT1699; FTT1344 | *pdpA* | hypothetical protein | 0.38 | tnfn1_pw060510p03q145 | NR-8065 | E06 |  | |  |
| FTN_1309 | FTT1699; FTT1344 | *pdpA* | hypothetical protein | 0.67 | tnfn1_pw060323p05q130 | NR-8039 | F04 |  | |  |
| FTN_1309 | FTT1699; FTT1344 | *pdpA* | hypothetical protein | 1.00 | tnfn1_pw060323p05q128 | NR-8039 | D04 |  | |  |
| **FTN_1310** | **FTT1700; FTT1345** | ***pdpB; icmF*** | **hypothetical protein** | **0.10** | **tnfn1_pw060323p05q179** | **NR-8039** | **G10** |  | |  |
| FTN_1310 | FTT1700; FTT1345 | *pdpB; icmF* | hypothetical protein | 0.44 | tnfn1_pw060418p02q165 | NR-8052 | A09 |  | |  |
| **FTN_1311** | **FTT1701; FTT1346** | ***iglE*** | **hypothetical protein** | **0.24** | **tnfn1_pw060328p01q194** | **NR-8043** | **F12** |  | |  |
| **FTN_1312** | **FTT1702; FTT1347** | ***vgrG*** | **hypothetical protein** | **0.25** | **tnfn1_pw060328p06q144** | **NR-8048** | **D06** |  | |  |
| FTN_1312 | FTT1702; FTT1347 | *vgrG* | hypothetical protein | 1.14 | tnfn1_pw060419p03q132 | NR-8057 | H04 |  | |  |
| **FTN_1313** | **FTT1703; FTT1348** | ***iglF*** | **hypothetical protein** | **0.28** | **tnfn1_pw060328p01q144** | **NR-8043** | **D06** |  | |  |
| FTN_1313 | FTT1703; FTT1348 | *iglF* | hypothetical protein | 0.60 | tnfn1_pw060328p04q154 | NR-8046 | F07 |  | |  |
| FTN_1313 | FTT1703; FTT1348 | *iglF* | hypothetical protein | 1.00 | tnfn1_pw060419p03q114 | NR-8057 | F02 |  | |  |
| **FTN_1314** | **FTT1704; FTT1349** | ***iglG*** | **hypothetical protein** | **0.10** | **tnfn1_pw060323p03q179** | **NR-8037** | **G10** |  | |  |
| FTN_1314 | FTT1704; FTT1349 | *iglG* | hypothetical protein | 0.60 | tnfn1_pw060418p04q141 | NR-8054 | A06 |  | |  |
| **FTN_1315** | **FTT1705; FTT1350** | ***iglH*** | **hypothetical protein** | **0.19** | **tnfn1_pw060328p06q163** | **NR-8048** | **G08** |  | |  |
| FTN_1315 | FTT1705; FTT1350 | *iglH* | hypothetical protein | 0.39 | tnfn1_pw060420p04q133 | NR-8062 | A05 |  | |  |
| **FTN_1316** | **FTT1706; FTT1351** | ***dotU*** | **hypothetical protein** | **0.17** | **tnfn1_pw060323p06q162** | **NR-8040** | **F08** |  | |  |
| FTN_1316 | FTT1706; FTT1351 | *dotU* | hypothetical protein | 0.31 | tnfn1_pw060419p01q125 | NR-8055 | A04 |  | |  |
| **FTN_1317** | **FTT1707; FTT1352** | ***iglI*** | **hypothetical protein** | **0.10** | **tnfn1_pw060323p02q142** | **NR-8036** | **B06** |  | |  |
| **FTN_1317** | **FTT1707; FTT1352** | ***iglI*** | **hypothetical protein** | **0.30** | **tnfn1_pw060420p04q177** | **NR-8062** | **E10** |  | |  |
| **FTN_1318** | **FTT1708; FTT1353** | ***iglJ*** | **hypothetical protein** | **0.12** | **tnfn1_pw060418p04q162** | **NR-8054** | **F08** |  | |  |
| **FTN_1318** | **FTT1708; FTT1353** | ***iglJ*** | **hypothetical protein** | **0.16** | **tnfn1_pw060328p06q148** | **NR-8048** | **H06** |  | |  |
| FTN_1319 | FTT1709; FTT1354 | *pdpC* | hypothetical protein | 0.50 | tnfn1_pw060323p01q171 | NR-8035 | G09 |  | |  |
| FTN_1319 | FTT1709; FTT1354 | *pdpC* | hypothetical protein | 1.33 | tnfn1_pw060418p04q106 | NR-8054 | F01 |  | |  |
| FTN_1320 | FTT1710; FTT1355 | *pdpE* | hypothetical protein | 0.50 | tnfn1_pw060323p03q140 | NR-8037 | H05 |  | |  |
| FTN_1320 | FTT1710; FTT1355 | *pdpE* | hypothetical protein | 0.88 | tnfn1_pw060420p01q177 | NR-8059 | E10 |  | |  |
| **FTN_1321** | **FTT1711c; FTT1356c** | ***iglD*** | **intracellular growth locus protein D** | **0.16** | **tnfn1_pw060510p01q110** | **NR-8063** | **B02** |  | |  |
| **FTN_1321** | **FTT1711c; FTT1356c** | ***iglD*** | **intracellular growth locus protein D** | **0.28** | **tnfn1_pw060420p03q137** | **NR-8061** | **E05** |  | |  |
| **FTN_1322** | **FTT1712c; FTT1357c** | ***iglC*** | **intracellular growth locus protein C** | **0.14** | **tnfn1_pw060418p02q116** | **NR-8052** | **H02** |  | |  |
| **FTN_1322** | **FTT1712c; FTT1357c** | ***iglC*** | **intracellular growth locus protein C** | **0.24** | **tnfn1_pw060328p06q115** | **NR-8048** | **G02** |  | |  |
| **FTN_1323** | **FTT1713c; FTT1358c** | ***iglB*** | **intracellular growth locus protein B** | **0.19** | **tnfn1_pw060328p02q115** | **NR-8044** | **G02** |  | |  |
| FTN_1323 | FTT1713c; FTT1358c | *iglB* | intracellular growth locus protein B | 0.45 | tnfn1_pw060419p01q141 | NR-8055 | A06 |  | |  |
| **FTN_1324** | **FTT1714c; FTT1359c** | ***iglA*** | **intracellular growth locus protein A** | **0.15** | **tnfn1_pw060323p05q159** | **NR-8039** | **C08** |  | |  |
| FTN_1324 | FTT1714c; FTT1359c | *iglA* | intracellular growth locus protein A | 0.57 | tnfn1_pw060420p03q125 | NR-8061 | A04 |  | |  |
| FTN_1325 | FTT1715c; FTT1360c | *pdpD* | hypothetical protein | 0.50 | tnfn1_pw060323p02q114 | NR-8036 | F02 |  | |  |
| FTN_1325 | FTT1715c; FTT1360c | *pdpD* | hypothetical protein | 0.89 | tnfn1_pw060419p04q108 | NR-8058 | H01 |  | |  |
| FTN_1326 | FTT1716c; FTT1361c | *anmK* | anhydro-N-acetylmuramic acid kinase | 1.00 | tnfn1_pw060328p06q183 | NR-8048 | C11 |  | |  |
| FTN_1326 | FTT1716c; FTT1361c | *anmK* | anhydro-N-acetylmuramic acid kinase | 1.17 | tnfn1_pw060420p03q122 | NR-8061 | F03 |  | |  |
| FTN_1357 | FTT1394c | *recB* | ATP-dependent exoDNAse (exonuclease V) beta subunit | 0.81 | tnfn1_pw060323p04q156 | NR-8038 | H07 |  | |  |
| FTN_1357 | FTT1394c | *recB* | ATP-dependent exoDNAse (exonuclease V) beta subunit | 1.00 | tnfn1_pw060323p08q169 | NR-8042 | E09 |  | |  |
| FTN_1357 | FTT1394c | *recB* | ATP-dependent exoDNAse (exonuclease V) beta subunit | 2.92 | tnfn1_pw060418p03q158 | NR-8053 | B08 |  | |  |
| FTN_1362 | FTT1400c | NA | hypothetical protein | 0.50 | tnfn1_pw060323p04q171 | NR-8038 | G09 |  | |  |
| FTN_1362 | FTT1400c | NA | hypothetical protein | 1.75 | tnfn1_pw060419p01q161 | NR-8055 | E08 |  | |  |
| FTN_1382 | FTT1416 | NA | hypothetical protein | 1.75 | tnfn1_pw060419p01q119 | NR-8055 | C03 |  | |  |
| FTN_1410 | FTT1441 | *bfr* | bacterioferritin | 1.17 | tnfn1_pw060420p03q191 | NR-8061 | C12 |  | |  |
| FTN_1410 | FTT1441 | *bfr* | bacterioferritin | 1.50 | tnfn1_pw060328p05q190 | NR-8047 | B12 |  | |  |
| FTN_1412 | FTT1442c | NA | DNA-directed RNA polymerase, alpha subunit/40 kD subunit | 0.50 | tnfn1_pw060323p04q184 | NR-8038 | D11 |  | |  |
| FTN_1417 | FTT1447c | *manB* | phosphomannomutase | 0.70 | tnfn1_pw060328p08q115 | NR-8050 | G02 |  | |  |
| FTN_1417 | FTT1447c | *manB* | phosphomannomutase | 1.67 | tnfn1_pw060510p03q186 | NR-8065 | F11 |  | |  |
| **FTN_1421** | **FTT1456c** | ***wbtH*** | **glutamine amidotransferase/asparagine synthase** | **0.15** | **tnfn1_pw060323p06q119** | **NR-8040** | **C03** |  | |  |
| FTN_1421 | FTT1456c | *wbtH* | glutamine amidotransferase/asparagine synthase | 0.56 | tnfn1_pw060420p04q116 | NR-8062 | H02 |  | |  |
| **FTN_1423** | **FTT1457c** | ***wbtG*** | **glycosyl transferase, group 1** | **0.15** | **tnfn1_pw060323p03q189** | **NR-8037** | **A12** |  | |  |
| FTN_1423 | FTT1457c | *wbtG* | glycosyl transferase, group 1 | 1.00 | tnfn1_pw060418p01q194 | NR-8051 | F12 |  | |  |
| FTN_1425 | FTT1459c | *wbtF* | NAD dependent epimerase | 0.33 | tnfn1_pw060323p06q161 | NR-8040 | E08 |  | |  |
| FTN_1425 | FTT1459c | *wbtF* | NAD dependent epimerase | 1.00 | tnfn1_pw060510p04q137 | NR-8066 | E05 |  | |  |
| FTN_1426 | FTT1460c | *wbtE* | UDP-glucose/GDP-mannose dehydrogenase family protein | 0.35 | tnfn1_pw060328p03q164 | NR-8045 | H08 |  | |  |
| FTN_1426 | FTT1460c | *wbtE* | UDP-glucose/GDP-mannose dehydrogenase family protein | 1.07 | tnfn1_pw060418p04q180 | NR-8054 | H10 |  | |  |
| **FTN_1427** | **FTT1461c** | ***wbtD*** | **glycosyl transferase, group 1** | **0.15** | **tnfn1_pw060323p01q134** | **NR-8035** | **B05** |  | |  |
| FTN_1427 | FTT1461c | *wbtD* | glycosyl transferase, group 1 | 1.00 | tnfn1_pw060510p03q107 | NR-8065 | G01 |  | |  |
| FTN_1427 | FTT1461c | *wbtD* | glycosyl transferase, group 1 | 1.17 | tnfn1_pw060419p04q192 | NR-8058 | D12 |  | |  |
| FTN_1431 | FTT1464c | *wbtA* | dTDP-glucose 4,6-dehydratase | 0.65 | tnfn1_pw060323p06q123 | NR-8040 | G03 |  | |  |
| FTN_1431 | FTT1464c | *wbtA* | dTDP-glucose 4,6-dehydratase | 0.75 | tnfn1_pw060419p03q166 | NR-8057 | B09 |  | |  |
| FTN_1433 | FTT1525c | NA | hypothetical protein | 1.00 | tnfn1_pw060323p05q154 | NR-8039 | F07 |  | |  |
| FTN_1433 | FTT1525c | NA | hypothetical protein | 1.11 | tnfn1_pw060420p02q142 | NR-8060 | B06 |  | |  |
| FTN_1438 | FTT1530 | NA | bifunctional protein: 3-hydroxacyl-CoA dehydrogenase/acyl-CoA-binding protein | 0.50 | tnfn1_pw060323p07q169 | NR-8041 | E09 |  |  | |
| FTN_1438 | FTT1530 | NA | bifunctional protein: 3-hydroxacyl-CoA dehydrogenase/acyl-CoA-binding protein | 1.28 | tnfn1_pw060418p02q122 | NR-8052 | F03 |  |  | |
| FTN_1470 | FTT1562 | *ispA* | geranyl diphosphate synthase/farnesyl diphosphate synthase | 0.75 | tnfn1_pw060419p04q138 | NR-8058 | F05 |  |  | |
| FTN_1470 | FTT1562 | *ispA* | geranyl diphosphate synthase/farnesyl diphosphate synthase | 0.89 | tnfn1_pw060328p04q118 | NR-8046 | B03 |  |  | |
| FTN_1471 | FTT1563 | *pcs* | (CDP-alcohol) phosphatidyltransferase | 0.71 | tnfn1_pw060323p06q184 | NR-8040 | D11 |  |  | |
| FTN_1500 | FTT1489 | NA | hypothetical protein | 0.89 | tnfn1_pw060510p02q135 | NR-8064 | C05 |  |  | |
| FTN_1500 | FTT1489 | NA | hypothetical protein | 1.00 | tnfn1_pw060328p06q151 | NR-8048 | C07 |  |  | |
| **FTN_1501** | **FTT1490** | **NA** | **monovalent cation:proton antiporter-1** | **0.22** | **tnfn1_pw060328p03q177** | **NR-8045** | **E10** |  |  | |
| FTN_1501 | FTT1490 | NA | monovalent cation:proton antiporter-1 | 1.00 | tnfn1_pw060419p03q196 | NR-8057 | H12 |  |  | |
| FTN_1502 | Intergenic (FTT1491c-1492c) | NA | hypothetical protein | 0.50 | tnfn1_pw060419p04q149 | NR-8058 | A07 |  |  | |
| FTN_1502 | Intergenic (FTT1491c-1492c) | NA | hypothetical protein | 0.72 | tnfn1_pw060323p04q133 | NR-8038 | A05 |  |  | |
| FTN_1513 | FTT1503 | *xerC* | site-specific recombinase | 0.75 | tnfn1_pw060328p06q169 | NR-8048 | E09 |  |  | |
| FTN_1513 | FTT1503 | *xerC* | site-specific recombinase | 1.25 | tnfn1_pw060510p03q193 | NR-8065 | E12 |  |  | |
| FTN_1518 | FTT1508c | *relA* | GDP pyrophosphokinase/GTP pyrophosphokinase | 0.50 | tnfn1_pw060323p06q110 | NR-8040 | B02 |  |  | |
| FTN_1518 | FTT1508c | *relA* | GDP pyrophosphokinase/GTP pyrophosphokinase | 0.50 | tnfn1_pw060323p07q167 | NR-8041 | C09 |  |  | |
| FTN_1538 | FTT1696 | *groEL* | chaperonin GroEL (HSP60 family) | 1.75 | tnfn1_pw060419p01q178 | NR-8055 | F10 |  |  | |
| FTN_1538 | FTT1696 | *groEL* | chaperonin GroEL (HSP60 family) | 1.50 | tnfn1_pw060328p05q129 | NR-8047 | E04 |  |  | |
| FTN_1548 | FTT0165c | NA | hypothetical protein | 0.67 | tnfn1_pw060420p03q194 | NR-8061 | F12 |  |  | |
| FTN_1548 | FTT0165c | NA | hypothetical protein | 0.72 | tnfn1_pw060328p01q101 | NR-8043 | A01 |  |  | |
| FTN_1548 | FTT0165c | NA | hypothetical protein | 0.89 | tnfn1_pw060323p06q150 | NR-8040 | B07 |  |  | |
| FTN_1551 | FTT0162 | *ampD* | N-acetylmuramoyl-L-alanine amidase | 1.36 | tnfn1_pw060328p06q177 | NR-8048 | E10 |  |  | |
| FTN_1551 | FTT0162 | *ampD* | N-acetylmuramoyl-L-alanine amidase | 2.17 | tnfn1_pw060510p04q129 | NR-8066 | E04 |  |  | |
| FTN_1558 | FTT0154 | *xerD* | site-specific recombinase | 0.65 | tnfn1_pw060418p03q117 | NR-8053 | A03 |  |  | |
| FTN_1558 | FTT0154 | *xerD* | site-specific recombinase | 0.68 | tnfn1_pw060328p01q175 | NR-8043 | C10 |  |  | |
| FTN_1582 | FTT0134 | NA | hypothetical membrane protein | 0.35 | tnfn1_pw060420p02q102 | NR-8060 | B01 |  |  | |
| FTN_1582 | FTT0134 | NA | hypothetical membrane protein | 0.59 | tnfn1_pw060323p08q185 | NR-8042 | E11 |  |  | |
| **FTN_1586** | **FTT0129** | **NA** | **sugar transporter, MFS superfamily** | **0.10** | **tnfn1_pw060418p01q150** | **NR-8051** | **B07** |  |  | |
| **FTN_1586** | **FTT0129** | **NA** | **sugar transporter, MFS superfamily** | **0.15** | **tnfn1_pw060328p01q110** | **NR-8043** | **B02** |  |  | |
| FTN_1597 | FTT0118 | *prfC* | peptide chain release factor 3 | 0.71 | tnfn1_pw060510p01q146 | NR-8063 | F06 |  |  | |
| FTN_1597 | FTT0118 | *prfC* | peptide chain release factor 3 | 0.81 | tnfn1_pw060328p01q106 | NR-8043 | F01 |  |  | |
| FTN_1602 | FTT0113 | *deoB* | phosphopentomutase | 0.50 | tnfn1_pw060323p04q137 | NR-8038 | E05 |  |  | |
| FTN_1602 | FTT0113 | *deoB* | phosphopentomutase | 1.00 | tnfn1_pw060418p01q174 | NR-8051 | B10 |  |  | |
| FTN_1607 | FTT0108c | *cca* | tRNA nucleotidyl transferase | 0.75 | tnfn1_pw060328p04q195 | NR-8046 | G12 |  |  | |
| **FTN_1608** | **FTT0107c** | ***dsbB*** | **disulfide bond formation protein** | **0.06** | **tnfn1_pw060510p02q157** | **NR-8064** | **A08** |  |  | |
| **FTN_1608** | **FTT0107c** | ***dsbB*** | **disulfide bond formation protein** | **0.20** | **tnfn1_pw060323p05q173** | **NR-8039** | **A10** |  |  | |
| FTN_1610 | FTT0105c | NA | RND efflux transporter, AcrB/AcrD/AcrF family | 0.50 | tnfn1_pw060323p02q131 | NR-8036 | G04 |  |  | |
| FTN_1610 | FTT0105c | NA | RND efflux transporter, AcrB/AcrD/AcrF family | 2.50 | tnfn1_pw060418p04q118 | NR-8054 | B03 |  |  | |
| FTN_1613 | FTT0101 | NA | peptidase, U61 family | 0.65 | tnfn1_pw060328p04q184 | NR-8046 | D11 |  |  | |
| FTN_1613 | FTT0101 | NA | peptidase, U61 family | 1.50 | tnfn1_pw060419p03q180 | NR-8057 | H10 |  |  | |
| FTN_1617 | FTT0094c | NA | two-component regulator, sensor histidine kinase | 0.66 | tnfn1_pw060323p08q137 | NR-8042 | E05 |  |  | |
| FTN_1617 | FTT0094c | NA | two-component regulator, sensor histidine kinase | 1.50 | tnfn1_pw060418p04q101 | NR-8054 | A01 |  |  | |
| FTN_1633 | FTT0078 | *apt* | adenine phosphoribosyltransferase | 0.50 | tnfn1_pw060323p01q166 | NR-8035 | B09 |  |  | |
| FTN_1633 | FTT0078 | *apt* | adenine phosphoribosyltransferase | 1.67 | tnfn1_pw060510p02q107 | NR-8064 | G01 |  |  | |
| FTN_1653 | FTT0057 | NA | hypothetical membrane protein | 1.00 | tnfn1_pw060328p05q181 | NR-8047 | A11 |  |  | |
| FTN_1654 | FTT0056c | NA | major facilitator superfamily (MFS) transport protein | 0.89 | tnfn1_pw060419p04q155 | NR-8058 | G07 |  |  | |
| FTN_1654 | FTT0056c | NA | major facilitator superfamily (MFS) transport protein | 0.90 | tnfn1_pw060328p08q189 | NR-8050 | A12 |  |  | |
| FTN_1655 | FTT0055 | *rluC* | ribosomal large subunit pseudouridine synthase C | 0.50 | tnfn1_pw060510p02q165 | NR-8064 | A09 |  |  | |
| FTN_1655 | FTT0055 | *rluC* | ribosomal large subunit pseudouridine synthase C | 1.17 | tnfn1_pw060323p04q160 | NR-8038 | D08 |  |  | |
| FTN_1656 | FTT0054 | NA | hypothetical protein | 0.85 | tnfn1_pw060420p02q127 | NR-8060 | C04 |  |  | |
| FTN_1656 | FTT0054 | NA | hypothetical protein | 1.00 | tnfn1_pw060328p08q182 | NR-8050 | B11 |  |  | |
| FTN_1657 | FTT0053 | NA | major facilitator superfamily (MFS) transport protein | 0.40 | tnfn1_pw060418p02q115 | NR-8052 | G02 |  |  | |
| FTN_1657 | FTT0053 | NA | major facilitator superfamily (MFS) transport protein | 0.64 | tnfn1_pw060328p02q171 | NR-8044 | G09 |  |  | |
| **FTN_1682** | **FTT0029c** | ***figA/ fslA*** | **siderophore biosynthesis protein** | **0.15** | **tnfn1_pw060323p04q131** | **NR-8038** | **G04** |  |  | |
| FTN_1682 | FTT0029c | *figA/ fslA* | siderophore biosynthesis protein | 0.64 | tnfn1_pw060419p04q180 | NR-8058 | H10 |  |  | |
| FTN_1682 | FTT0029c | *figA/ fslA* | siderophore biosynthesis protein | 0.70 | tnfn1_pw060328p05q150 | NR-8047 | B07 |  |  | |
| **FTN_1683** | **FTT0028c** | ***figB/ fslB*** | **Conserved membrane protein** | **0.15** | **tnfn1_pw060323p03q163** | **NR-8037** | **G08** |  |  | |
| **FTN_1683** | **FTT0028c** | ***figB/ fslB*** | **Conserved membrane protein** | **0.18** | **tnfn1_pw060419p04q105** | **NR-8058** | **E01** |  |  | |
| **FTN_1683** | **FTT0028c** | ***figB/ fslB*** | **Conserved membrane protein** | **0.20** | **tnfn1_pw060418p02q193** | **NR-8052** | **E12** |  |  | |
| **FTN_1683** | **FTT0028c** | ***figB/ fslB*** | **Conserved membrane protein** | **0.20** | **tnfn1_pw060328p02q192** | **NR-8044** | **D12** |  |  | |
| **FTN_1684** | **FTT0027c** | ***figC/ fslC*** | **diaminopimelate decarboxylase** | **0.24** | **tnfn1_pw060510p02q193** | **NR-8064** | **E12** |  |  | |
| FTN_1684 | FTT0027c | *figC/ fslC* | **diaminopimelate decarboxylase** | 0.40 | tnfn1_pw060328p03q166 | NR-8045 | B09 |  |  | |
| FTN_1684 | FTT0027c | *figC/ fslC* | **diaminopimelate decarboxylase** | 1.00 | tnfn1_pw060510p02q191 | NR-8064 | C12 |  |  | |
| **FTN_1699** | **FTT1720c** | ***purL*** | **phosphoribosylformylglycinamide synthase** | **0.11** | **tnfn1_pw060510p03q121** | **NR-8065** | **E03** |  |  | |
| **FTN_1699** | **FTT1720c** | ***purL*** | **phosphoribosylformylglycinamide synthase** | **0.12** | **tnfn1_pw060323p06q112** | **NR-8040** | **D02** |  |  | |
| **FTN_1700** | **FTT1720c** | ***purL*** | **phosphoribosylformylglycinamide synthase** | **0.17** | **tnfn1_pw060419p01q190** | **NR-8055** | **B12** |  |  | |
| FTN_1705 | FTT1726 | NA | peptidase, U32 family | 0.83 | tnfn1_pw060420p04q131 | NR-8062 | G04 |  |  | |
| FTN_1705 | FTT1726 | NA | peptidase, U32 family | 1.17 | tnfn1_pw060328p05q105 | NR-8047 | E01 |  |  | |
| **FTN_1715** | **FTT1736c** | ***kdpD*** | **two component regulator, sensor histidine kinase kdpD** | **0.11** | **tnfn1_pw060328p01q124** | **NR-8043** | **H03** |  |  | |
| FTN_1715 | FTT1736c | *kdpD* | two component regulator, sensor histidine kinase kdpD | 0.68 | tnfn1_pw060510p03q184 | NR-8065 | D11 |  |  | |
| FTN_1715 | FTT1736c | *kdpD* | two component regulator, sensor histidine kinase kdpD | 1.75 | tnfn1_pw060419p01q150 | NR-8055 | B07 |  |  | |
| **FTN_1743** | **FTT1769c** | ***clpB*** | **chaperone clpB** | **0.29** | **tnfn1_pw060420p01q140** | **NR-8059** | **H05** |  |  | |
| FTN_1743 | FTT1769c | *clpB* | chaperone clpB | 1.07 | tnfn1_pw060510p02q130 | NR-8064 | F04 |  |  | |
| FTN_1743 | FTT1769c | *clpB* | chaperone clpB | 1.17 | tnfn1_pw060328p03q122 | NR-8045 | F03 |  |  | |
| FTN_1744 | FTT1768c | *chiB* | chitinase | 0.71 | tnfn1_pw060328p03q184 | NR-8045 | D11 |  |  | |
| FTN_1744 | FTT1768c | *chiB* | chitinase | 1.39 | tnfn1_pw060419p04q131 | NR-8058 | G04 |  |  | |
| FTN_1745 | FTT1767c | *purT* | phosphoribosylglycinamide formyltransferase 2 | 0.78 | tnfn1_pw060510p01q164 | NR-8063 | H08 |  |  | |
| FTN_1745 | FTT1767c | *purT* | phosphoribosylglycinamide formyltransferase 2 | 0.90 | tnfn1_pw060328p08q169 | NR-8050 | E09 |  |  | |
| FTN_1745 | FTT1767c | *purT* | phosphoribosylglycinamide formyltransferase 2 | 1.00 | tnfn1_pw060510p01q133 | NR-8063 | A05 |  |  | |
| FTN_1750 | FTT1762c | NA | acyltransferase | 0.63 | tnfn1_pw060510p02q173 | NR-8064 | A10 |  |  | |
| FTN_1750 | FTT1762c | NA | acyltransferase | 0.89 | tnfn1_pw060328p02q134 | NR-8044 | B05 |  |  | |
| FTN_1750 | FTT1762c | NA | acyltransferase | 1.50 | tnfn1_pw060418p03q116 | NR-8053 | H02 |  |  | |
| FTN_1750 | FTT1762c | NA | acyltransferase | 2.50 | tnfn1_pw060419p03q128 | NR-8057 | D04 |  |  | |
| FTN_1762 | FTT1782c | NA | (putative) drug resistance ATPase-1 (Drug RA1) family protein | 1.00 | tnfn1_pw060419p04q170 | NR-8058 | F09 |  |  | |
| FTN_1762 | FTT1782c | NA | (putative) drug resistance ATPase-1 (Drug RA1) family protein | 1.07 | tnfn1_pw060328p04q169 | NR-8046 | E09 |  |  | |

**Table S1 References**

1. Weiss DS, Brotcke A, Henry T, Margolis JJ, Chan K, et al. (2007) In vivo negative selection screen identifies genes required for Francisella virulence. Proc Natl Acad Sci U S A 104: 6037-6042.

2. Qin A, Mann BJ (2006) Identification of transposon insertion mutants of Francisella tularensis tularensis strain Schu S4 deficient in intracellular replication in the hepatic cell line HepG2. BMC Microbiol 6: 69.

3. Schulert GS, McCaffrey RL, Buchan BW, Lindemann SR, Hollenback C, et al. (2009) Francisella tularensis genes required for inhibition of the neutrophil respiratory burst and intramacrophage growth identified by random transposon mutagenesis of strain LVS. Infect Immun 77: 1324-1336.

4. Asare R, Abu Kwaik Y (2010) Molecular complexity orchestrates modulation of phagosome biogenesis and escape to the cytosol of macrophages by Francisella tularensis. Environ Microbiol.

5. Ahlund MK, Ryden P, Sjostedt A, Stoven S (2010) A directed screen of Francisella novicida virulence determinants using Drosophila melanogaster. Infect Immun.

6. Tempel R, Lai XH, Crosa L, Kozlowicz B, Heffron F (2006) Attenuated Francisella novicida transposon mutants protect mice against wild-type challenge. Infect Immun 74: 5095-5105.

7. Ahlund MK, Ryden P, Sjostedt A, Stoven S (2010) Directed screen of Francisella novicida virulence determinants using Drosophila melanogaster. Infect Immun 78: 3118-3128.

8. Maier TM, Casey MS, Becker RH, Dorsey CW, Glass EM, et al. (2007) Identification of Francisella tularensis Himar1-based transposon mutants defective for replication in macrophages. Infect Immun 75: 5376-5389.

9. Lindemann SR, Peng K, Long ME, Hunt JR, Apicella MA, et al. (2011) Francisella tularensis Schu S4 O-antigen and capsule biosynthesis gene mutants induce early cell death in human macrophages. Infect Immun 79: 581-594.

10. Su J, Yang J, Zhao D, Kawula TH, Banas JA, et al. (2007) Genome-wide identification of Francisella tularensis virulence determinants. Infect Immun 75: 3089-3101.

11. Wehrly TD, Chong A, Virtaneva K, Sturdevant DE, Child R, et al. (2009) Intracellular biology and virulence determinants of Francisella tularensis revealed by transcriptional profiling inside macrophages. Cell Microbiol 11: 1128-1150.

12. Kraemer PS, Mitchell A, Pelletier MR, Gallagher LA, Wasnick M, et al. (2009) Genome-wide screen in Francisella novicida for genes required for pulmonary and systemic infection in mice. Infect Immun 77: 232-244.

13. Quarry JE, Isherwood KE, Michell SL, Diaper H, Titball RW, et al. (2007) A Francisella tularensis subspecies novicida purF mutant, but not a purA mutant, induces protective immunity to tularemia in mice. Vaccine 25: 2011-2018.

14. Kadzhaev K, Zingmark C, Golovliov I, Bolanowski M, Shen H, et al. (2009) Identification of genes contributing to the virulence of Francisella tularensis SCHU S4 in a mouse intradermal infection model. PLoS One 4: e5463.

15. Pechous R, Celli J, Penoske R, Hayes SF, Frank DW, et al. (2006) Construction and characterization of an attenuated purine auxotroph in a Francisella tularensis live vaccine strain. Infect Immun 74: 4452-4461.

16. Kanistanon D, Hajjar AM, Pelletier MR, Gallagher LA, Kalhorn T, et al. (2008) A Francisella mutant in lipid A carbohydrate modification elicits protective immunity. PLoS Pathog 4: e24.

17. Qin A, Scott DW, Thompson JA, Mann BJ (2009) Identification of an essential Francisella tularensis subsp. tularensis virulence factor. Infect Immun 77: 152-161.

18. Nano FE, Zhang N, Cowley SC, Klose KE, Cheung KK, et al. (2004) A Francisella tularensis pathogenicity island required for intramacrophage growth. J Bacteriol 186: 6430-6436.

19. Lauriano CM, Barker JR, Yoon SS, Nano FE, Arulanandam BP, et al. (2004) MglA regulates transcription of virulence factors necessary for Francisella tularensis intraamoebae and intramacrophage survival. Proc Natl Acad Sci U S A 101: 4246-4249.

20. Brotcke A, Weiss DS, Kim CC, Chain P, Malfatti S, et al. (2006) Identification of MglA-regulated genes reveals novel virulence factors in Francisella tularensis. Infect Immun 74: 6642-6655.

21. Schmerk CL, Duplantis BN, Howard PL, Nano FE (2009) A Francisella novicida pdpA mutant exhibits limited intracellular replication and remains associated with the lysosomal marker LAMP-1. Microbiology 155: 1498-1504.

22. Barker JR, Chong A, Wehrly TD, Yu JJ, Rodriguez SA, et al. (2009) The Francisella tularensis pathogenicity island encodes a secretion system that is required for phagosome escape and virulence. Mol Microbiol 74: 1459-1470.

23. Gray CG, Cowley SC, Cheung KK, Nano FE (2002) The identification of five genetic loci of Francisella novicida associated with intracellular growth. FEMS Microbiol Lett 215: 53-56.

24. Santic M, Molmeret M, Barker JR, Klose KE, Dekanic A, et al. (2007) A Francisella tularensis pathogenicity island protein essential for bacterial proliferation within the host cell cytosol. Cell Microbiol 9: 2391-2403.

25. Golovliov I, Sjostedt A, Mokrievich A, Pavlov V (2003) A method for allelic replacement in Francisella tularensis. FEMS Microbiol Lett 222: 273-280.

26. Lai XH, Golovliov I, Sjostedt A (2004) Expression of IglC is necessary for intracellular growth and induction of apoptosis in murine macrophages by Francisella tularensis. Microb Pathog 37: 225-230.

27. Cong Y, Yu JJ, Guentzel MN, Berton MT, Seshu J, et al. (2009) Vaccination with a defined Francisella tularensis subsp. novicida pathogenicity island mutant (DeltaiglB) induces protective immunity against homotypic and heterotypic challenge. Vaccine 27: 5554-5561.

28. de Bruin OM, Ludu JS, Nano FE (2007) The Francisella pathogenicity island protein IglA localizes to the bacterial cytoplasm and is needed for intracellular growth. BMC Microbiol 7: 1.

29. Ludu JS, de Bruin OM, Duplantis BN, Schmerk CL, Chou AY, et al. (2008) The Francisella pathogenicity island protein PdpD is required for full virulence and associates with homologues of the type VI secretion system. J Bacteriol 190: 4584-4595.

30. Raynaud C, Meibom KL, Lety MA, Dubail I, Candela T, et al. (2007) Role of the wbt locus of Francisella tularensis in lipopolysaccharide O-antigen biogenesis and pathogenicity. Infect Immun 75: 536-541.

31. Bell BL, Mohapatra NP, Gunn JS (2010) Regulation of Virulence Gene Transcripts by the Francisella Orphan Response Regulator PmrA: Role of Phosphorylation and Evidence of MglA/SspA Interaction. Infect Immun.

32. Meibom KL, Dubail I, Dupuis M, Barel M, Lenco J, et al. (2008) The heat-shock protein ClpB of Francisella tularensis is involved in stress tolerance and is required for multiplication in target organs of infected mice. Mol Microbiol 67: 1384-1401.

33. Conlan JW, Shen H, Golovliov I, Zingmark C, Oyston PC, et al. (2010) Differential ability of novel attenuated targeted deletion mutants of Francisella tularensis subspecies tularensis strain SCHU S4 to protect mice against aerosol challenge with virulent bacteria: effects of host background and route of immunization. Vaccine 28: 1824-1831.

34. Gallagher LA, Ramage E, Jacobs MA, Kaul R, Brittnacher M, et al. (2007) A comprehensive transposon mutant library of Francisella novicida, a bioweapon surrogate. Proc Natl Acad Sci U S A 104: 1009-1014.
