# Supplementary material for: Macrophage Replication Screen Identifies a Novel Francisella Hydroperoxide Resistance Protein Involved in Virulence
Source: PLoS One. 2011 Sep 6;6(9):e24201. doi: 10.1371/journal.pone.0024201 (PMC3167825; doi:10.1371/journal.pone.0024201)
Supplement: Table S2 — Primers used in this study. (DOC) [file pone.0024201.s004.doc]

| **Primer name** | **Sequence** |
| --- | --- |
| **FTN_0096** |  |
| 96 deletion check F1 | accctctaatgtaatatcgc |
| 96 Arm1 FWD | caattttccatggctgagta |
| 96 Arm 1 REV | ttatcgataccgtcgacctcttttttactctcctcttttagc |
| 96 frt_sKAN_frt FWD | gctaaaagaggagagtaaaaaagaggtcgacggtatcgataa |
| 96 frt_sKAN_frt REV | actctaaattaaaggctatgatgcatagctgcaggatcgata |
| 96 Arm 2 FWD | tatcgatcctgcagctatgcatcatagcctttaatttagagt |
| 96 Arm 2 REV | caattataataggcagctcc |
| 96 deletion check R1 | cagcgtttccaatactacta |
| 96 comp Arm 1 REV | gagtgacaacccaaagagatttaaaggctatgatttttaactt |
| 96 comp sKAN FWD | aagttaaaaatcatagcctttaaatctctttgggttgtcactc |
| 96 comp sKAN REV | atttattaccaactctaaattaaaggctatgatacaaccaattaaccaattctg |
| 96 comp Arm 2 FWD | cagaattggttaattggttgtatcatagcctttaatttagagttggtaataaat |
| 96 comp check R2 | gggaacttaagaattctagg |
|  |  |
| **FTN_1133** |  |
| 1133 deletion check F1 | ctttctctaacttgtgcctt |
| 1133 Arm1 FWD | ataggatattctcctgagtg |
| 1133 Arm 1 REV | ttatcgataccgtcgacctctagattttaatccttataatatttt |
| 1133 frt_sKAN_frt FWD | aaaatattataaggattaaaatctagaggtcgacggtatcgataa |
| 1133 frt_sKAN_frt REV | tgagatatttaaacttattatttaagcatagctgcaggatcgata |
| 1133 Arm 2 FWD | tatcgatcctgcagctatgcttaaataataagtttaaatatctca |
| 1133 Arm 2 REV | acttttctcattaccttggc |
| 1133 deletion check R1 | gggctagagctattttgaat |
| 1133 comp Arm 1 REV | gagtgacaacccaaagagatttagcttttattatcgatcaag |
| 1133 comp sKAN FWD | cttgatcgataataaaagctaaatctctttgggttgtcactc |
| 1133 comp sKAN REV | tgagatatttaaacttattatttaaacaaccaattaaccaattctg |
| 1133 comp Arm 2 FWD | cagaattggttaattggttgtttaaataataagtttaaatatctca |
| 1133 comp check R2 | cagatgaatggtcaactctt |
|  |  |
| **FTL_0803** |  |
| **TargeTron primers** |  |
| FTL_0803 192/3a-IBS | aaaactcgagataattatccttaacaatctgactgacttgtgcgcccagatagggt |
| FTL_0803 192/3a-EBS1d | cagattgtacaaatgtggtgataacagataagtctgacttagtaacttacctttctttgt |
| FTL_0803 192/3a-EBS2 | tgaacgcaagtttctaatttcgattattgttcgatagaggaaagtgtct |
| **Clean deletion primers** |  |
| FTL_0803 Arm 1 FWD | atatatatggatccgtcatactcgatcataaacg |
| FTL_0803 Arm 1 REV | gagatatttaaacttattatttaatagattttaatccttataatattttttg |
| FTL_0803 Arm 2 FWD | caaaaaatattataaggattaaaatctattaaataataagtttaaatatctc |
| FTL_0803 Arm 2 REV | atatatatGGATCCgattaaagctcaagtcgttg |
| FTL_0803 Check F1 | gatgcttttccataccaaca |
| FTL_0803 Check F2 | gcaagatcttcatgcatataac |
| FTL_0803 Check R1 | gaatatattgctggtatggc |
|  |  |
| **RT Primers** |  |
| uvrD RT1 | gggatgtcgccttttgattttc |
| uvrD RT2 | ctcttttgtcccttgtgcttgc |
| FTN_1133 RT3 | gatattcgttgtttagcttgc |
| FTN_1133 RT5 | ggtcaactcttctcaatgag |
